# SeekDeep: single-base resolution *de novo* clustering for amplicon deep sequencing

**DOI:** 10.1093/nar/gkx1201

**Published:** 2017-11-30

**Authors:** Nicholas J Hathaway, Christian M Parobek, Jonathan J Juliano, Jeffrey A Bailey

**Affiliations:** 1 Program in Bioinformatics and Integrative Biology, University of Massachusetts Medical School, Worcester, MA, USA; 2 Curriculum in Genetics and Molecular Biology, University of North Carolina School of Medicine, Chapel Hill, NC, USA; 3 Division of Infectious Diseases, Department of Medicine, University of North Carolina, Chapel Hill, NC, USA; 4 Division of Transfusion Medicine, Department of Medicine, University of Massachusetts Medical School, Worcester, MA, USA

## Abstract

PCR amplicon deep sequencing continues to transform the investigation of genetic diversity in viral, bacterial, and eukaryotic populations. In eukaryotic populations such as *Plasmodium falciparum* infections, it is important to discriminate sequences differing by a single nucleotide polymorphism. In bacterial populations, single-base resolution can provide improved resolution towards species and strains. Here, we introduce the SeekDeep suite built around the qluster algorithm, which is capable of accurately building *de novo* clusters representing true, biological local haplotypes differing by just a single base. It outperforms current software, particularly at low frequencies and at low input read depths, whether resolving single-base differences or traditional OTUs. SeekDeep is open source and works with all major sequencing technologies, making it broadly useful in a wide variety of applications of amplicon deep sequencing to extract accurate and maximal biologic information.

## INTRODUCTION

The development of targeted next-generation sequencing technologies has dramatically expanded research into population-level genetic diversity, from the study of bacterial communities (1), intrahost variation in infections, such as HIV and malaria (2–4), to heterogeneity in cancer tumors (5). In general, targeted amplicon deep sequencing utilizes areas of conserved sequence for amplification primer placement, surrounding a region of interest containing known mutations or high sequence variability. Thousands to millions of product molecules from the amplification are then individually sequenced using current massively parallel techniques. To date, experimental and computational techniques for deep sequencing have been driven largely by microbiome 16S and targeted viral sequencing where single-base resolution is not a necessity (2,6,7). While initial microbiome work has focused on genus-level resolution of 97% sequence identity, there is greater interest in maximizing species and strain information in bacterial and viral populations (8,9). In eukaryotic populations, such as malaria strains, and for mutation detection, differentiation at the single-nucleotide level resolution is a necessity (3,4).

The central bioinformatic challenge of all targeted deep sequencing is to accurately resolve the true biologic differences that are obscured by the numerous errors introduced during PCR amplification and sequencing. PCR errors include substitutions, insertions and deletions, as well as chimeras formed by incomplete extension and subsequent re-priming on a highly-similar (but non-identical) template (Supplementary Figure S1). Sequencing error types and frequencies tend to be platform specific, and are related to either the sequencing polymerase or detection technology. For instance, pyrosequencing-based technologies generate numerous insertion-deletion (indel) errors, particularly in homopolymers, since these technologies estimate the number of a particular nucleotide in succession based on the cumulative fluorescent (454) or ion (Ion Torrent) signal. On the other hand, Illumina technology mainly misidentifies individual nucleotides, thus producing base-substitution errors (10,11).

Numerous computational solutions have been developed to correct for these errors including MetAmp(12), minimum entropy decomposition (MED (13)), homopolymer runs correction (Acacia (14)), clustering based on consistency of inferred error models (DADA2 (15)), operational taxonomic unit (OTU) clustering (UPARSE (16)), k-mer correcting (KEC (17)), and many others (18,19). All of these methods have advantages and disadvantages vis-a-vis speed, sensitivity, specificity, flexibility, range of sequencing technologies, and types of errors corrected. In general, the latest methods aim for greater resolution to allow better definition of microbial populations. The ultimate goal is discriminating sequences differing by a single base, which is the quantum level of evolutionary change. Such resolution will allow more detailed assessment of bacterial, viral, and eukaryotic microbial populations particularly with longer amplicons. Consistent single-base resolution is a particular necessity for studies of eukaryotic intra-species populations and for mutation detection. For example, in malaria research, the sequence of a single amplicon is frequently used to define strains within an infected individual, and these sequences often differ by only a single base, representative of a SNP within the larger parasite population. In microbiome studies, single-base resolution of 16S amplicon clustering extracts maximal information for downstream analyses. Thus, we sought to develop new algorithms that could consistently differentiate single-base differences in a wide variety of conditions and applications including improved accuracy and sensitivity of traditional operational taxonomic units (OTUs).

Here, we present SeekDeep, an open-source software suite for *de novo* (i.e reference free) analysis of amplicons that is fast, sensitive, customizable, and is able to resolve sequences differing by only a single base, even at low frequencies. At the center of SeekDeep is the algorithm qluster (for quality clustering) that improves the correction of PCR and sequencing errors in multiple key ways including base quality values and k-mer frequencies. SeekDeep also provides a growing set of pre- and post-processing tools, including an embedded web server to dynamically view results and ancillary data - particularly useful when working with large datasets and numerous samples, a scenario which has become common with targeted amplicon studies (3,4).

We compared SeekDeep to other recent best-in-class programs, DADA2 (15), MED (13) and UNOISE in USEARCH (preprint https://doi.org/10.1101/081257), which also aim for single-base resolution. All programs aim to determine the local PCR amplicon haplotypes, herein referred to simply as haplotypes for brevity, that represent the specific sequences (linked variation from the same chromosome) found in the biologic material prior to amplification. We also compared OTU based clustering to commonly used programs USEARCH (aka UCLUST/UPARSE) (16) and to Swarm (20) which cannot resolve at the single-base level. We focused our comparisons on programs that could work with both Illumina and 454/Ion Torrent sequence and did not compare to programs that only correct 454 and Ion Torrent pyrosequencing errors like AmpliconNoise (6), Acacia (14), and HECTOR (21) as we are interested in tools that are broadly applicable in the field.

To ascertain the performance of these programs, we compared results of *in silico* simulated datasets and *in vitro* mixtures of isolated DNA representing mock infections of both *Plasmodium falciparum* and bacterial communities. The simulations focused on the quantitative accuracy of discerning minor (low-abundance) haplotypes in terms of how much they differ (1–13 bp equating to 99.6–95.6% similarity) from a major (high-abundance) haplotype and how much they differ from another minor haplotype unrelated to all other haplotypes.

## MATERIALS AND METHODS

### Overview of the SeekDeep suite

**Figure 1.**
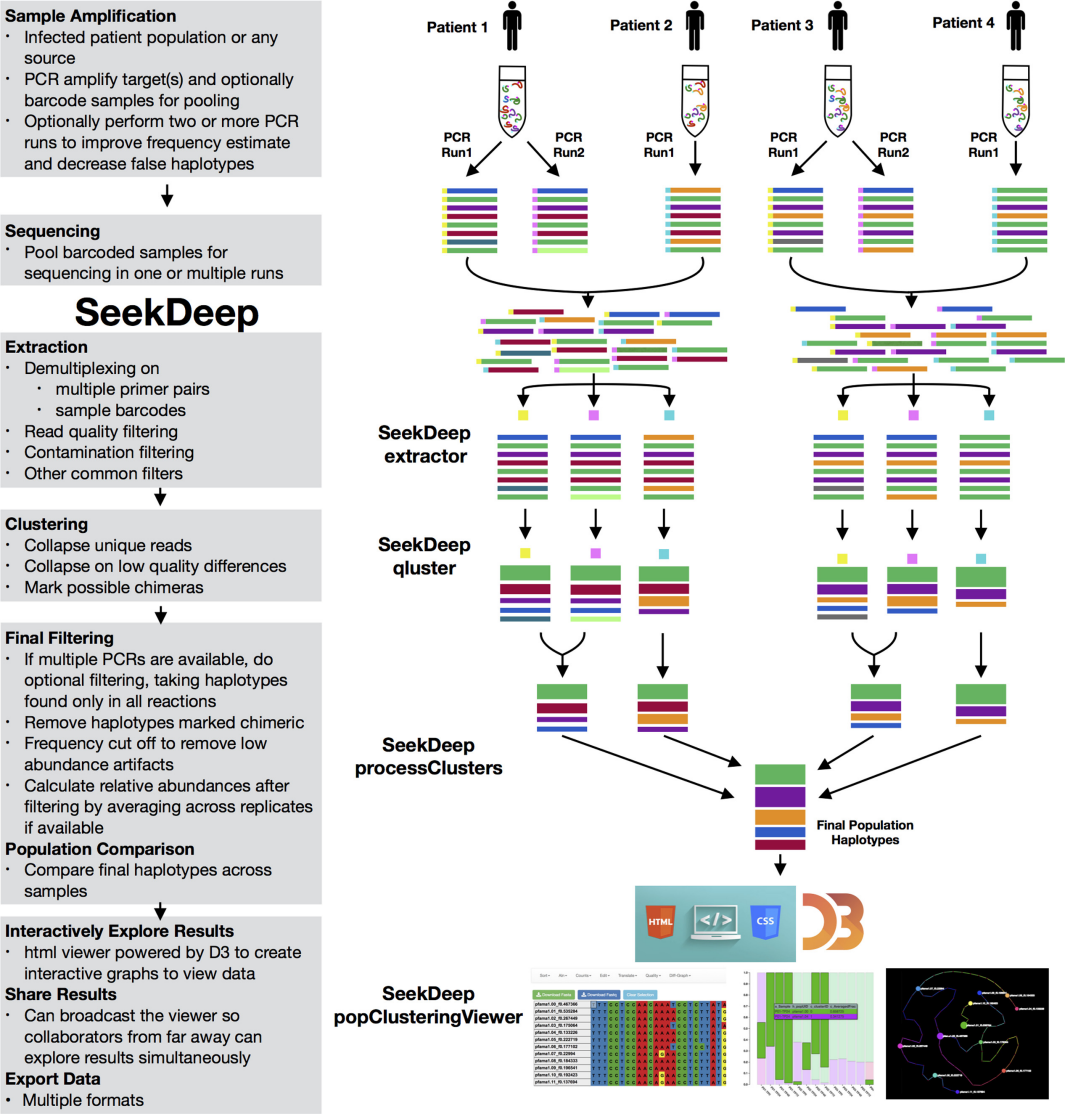
SeekDeep Overview. The depicted SeekDeep pipeline was designed to handle diverse experimental and computational workflows. In general, input sequence data is organized as one or more groups of samples that can represent natural populations, different experimental conditions, or any other defined classification. The pipeline is modular, allowing for substitute or additional processing at any step as well as access to the underlying data. The goal of SeekDeep is to perform initial processing and clustering along with exploration of the results and quality control. Extraction is done by **extractor** to demultiplex on sample barcodes (depicted here as colored squares at the beginning of sequences) and/or multiple primers if either are still present in input data. Next, sequences are clustered at the sample level by **qluster** based on either presets for specific sequencing technologies or user defined parameters to provide the requisite level of resolution (see Supplementary Figure S2 for how these errors are characterized). Finally the haplotypes generated by **qluster** are analyzed by **processClusters** to take into account replicate comparisons (if available) and then compare sample haplotypes to generate population-level haplotypes and statistics. Final results can be viewed with **popClusteringViewer** in an interactive HTML viewer. For more specific downstream analyses, data can be outputted in multiple formats.

SeekDeep is a software suite written in C++ centered around *de novo* clustering providing rapid sample and input sequence preprocessing, and postprocessing sample and population summaries for further downstream analysis. SeekDeep can be utilized with most major sequencing technologies, including Ion Torrent, 454 and Illumina, to swiftly analyze numerous samples and amplicons (Figure 1). SeekDeep provides start-to-finish workflow from raw sequence files to population-level clustering and tabular and graphical summaries. SeekDeep is freely available under the GNU Lesser General Public License v3.0 and is actively developed on github (https://github.com/bailey-lab/SeekDeep) while usage and details on the program can be found at the SeekDeep website (http://baileylab.umassmed.edu/SeekDeep/). SeekDeep has three main components, extractor, qluster and processClusters, that are central to generating clustering results, and an additional component, popClusteringViewer, to aid in viewing and sharing the results.

### extractor: de-multiplexing and read filtering

The subprogram extractor is generalized to process 454 and Ion Torrent standard flowgram format (SFF) files and standard FASTQ files from any source. Extractor also demultiplexes samples and amplicons using a wide variety of barcode and primer schemes but can also operate on already demultiplexed data (e.g. data that has been demultiplexed by standard Illumina pipelines). Like most extraction programs, SeekDeep includes typical tools for initial filtering based on read length, presence of primers, quality score metrics and/or presence of ambiguous bases (i.e. Ns). Extractor first separates reads based on sample barcodes handling a wide range of barcoding schemes that are commonly employed. Next, multiple or a single pair of forward and reverse primers are detected, demultiplexed and removed. Filtering is then done on per base quality scores, and on expected read lengths which can be set per primer set. Also, optional contamination filtering can be performed by supplying the sequences of target regions whereby sequences that differ drastically from these are removed.

See http://baileylab.umassmed.edu/SeekDeep/extractor_usage for full details on the options offered by extractor.

### qluster: rapid and accurate clustering based on quality

At the core of the SeekDeep package is the qluster algorithm, which iteratively collapses amplicon reads based on pairwise global alignments (Supplementary Figures S2 and S3). It leverages sequencer-generated quality values to discern likely true differences from sequencing errors as well as k-mer frequencies to filter out likely low abundance PCR errors. Although SeekDeep can process multiple amplicons at once, they are processed independently and haplotypes are not built or phased across different amplicons. The clustering process is summarized below.

First, reads lacking differences are collapsed into identical sequence clusters, which are then indexed for k-mers (default size 9). These initial identical clusters are then sorted based on the associated number of reads. An iterative comparison process is then undertaken with successive rounds of clustering allowing for an increasing number of differences to trigger the merger of two clusters. Majority-rule consensus of the smallest clusters are pairwise aligned and compared sequentially to the consensus of the largest clusters to determine if they should be merged into one cluster or remain as two separate clusters (Supplementary Figures S2 and S3). Once the clusters have all been initially compared and collapsed, if meeting threshold, the threshold for collapse is stepwise-raised to allow for more divergence in a subsequent iteration. At the end of each iteration majority-rule consensus are generated to represent each of the clusters. If consensus have changed due to the addition of new sequences, the clusters are again compared at the same error thresholds before advancing to the next iteration. The algorithm allows for flexibility not only in the number of iterations, but also in the threshold number and type of differences to collapse. Differences are classified as one-base indels, two-base indels, greater than two-base indels, low-quality mismatches, high-quality mismatches, and low k-mer frequency mismatches. In this way, the clustering is similar to operational taxonomic unit (OTU) percent identity clustering, but instead of counting all differences equally we are able to weigh the type and the quality of the difference before determining whether to merge clusters—an important feature for sequencing technology-aware clustering.

For clustering iterations, there are default collapse threshold profiles for 454, Ion Torrent, and Illumina, or a custom file can be supplied. The custom input parameter file allows the expert user to balance sensitivity, specificity, and speed for specific applications. The default profiles were used for all analyses in this paper. For a 454/Ion Torrent dataset, our standard error profile limits initial collapsing to sequences differing by single-base indels, given that the predominant errors in these datasets are small indels caused by homopolymer misestimation. On an Illumina dataset, which is unlikely to have erroneous single-base indels but more likely to have base miscalls, the default profile does not collapse on indels but allows more low quality mismatches. This framework makes the qluster algorithm highly extensible and adaptable to changing error profiles in updated or novel sequencing platforms. In terms of applications, the ability to collapse to an exact number of differences allows for biologic questions to be concretely addressed. For example, settings could allow 2–3 mismatches when sequencing viruses like HIV to collapse the viral clouds, or settings could be used to not allow a single high quality difference when searching for point mutations in the domain of a gene associated with drug resistance.

#### Differentiating mismatches with quality

The quality of any mismatch is determined by assessing the quality scores of the two mismatching bases in the pairwise alignment between clusters and the quality of the neighboring bases in the region (22). A primary quality and a neighboring quality is calculated. For a mismatch to be considered high quality, it must exceed the set thresholds for both of these quality values. The number of neighboring bases included can be changed; the default value is 2, which includes two bases upstream and downstream for a total of four neighboring bases examined. If a mismatch is determined to be a high quality error, its *k*-mer frequency is also checked to determine if the mismatch is in a low frequency *k*-mer. To calculate this, the mismatched base is centered in odd number length *k*-mer (defaulting to 9). Next, the previously indexed k-mers are checked to determine if mismatched centered *k*-mer has a low frequency—either as user defined or as a percentage of total reads. The *k*-mer cutoff defaults to 1 read, so if the *k*-mer occurs only once in the sample read set it is counted as a low frequency error. The *k*-mer position within the sequence can also be taken into account and helps to improve the filtering when repeats are present.

#### Homopolymer indel weighting for 454 and Ion Torrent

In the Ion Torrent and 454 technologies, the most common errors are indels in homopolymers. Thus, for homopolymers, indels are weighted to count less than other indels rather than separately categorizing them. Weighting incorporates the length by taking the size of the indel and dividing by the average size of both homopolymer runs. For example, a single-base indel found in a homopolymer of four bases (meaning one read has four bases and the other has three bases), the indel weight will be counted as 1/3.5 instead of 1.

#### Chimera detection

After clustering, the resultant haplotypes can be examined for likely chimeras that may have resulted from PCR (Supplementary Figure S1B). If replicates are available, then potential chimeras not appearing in all replicates will be removed. However, chimeras are often reproducible (23) which requires additional checks. This is accomplished by pairwise comparison of all the putative haplotypes from qluster checking to see if any cluster could be the result of a composite of two other clusters, which is similar to other approaches (6,15). Since, by definition, parental haplotypes contributing to a cluster must preexist for a chimera to form from them, we normally require that the parents are of equal or greater abundance relative to the potential chimera. By default, chimeras are called when both parents are at least 2-fold greater in abundance (user definable). This is a conservative approach to minimize false discovery that prioritizes removal of artifactual chimeras at the cost of potentially excluding low abundance biologic recombinants, but for most applications chimeras tend to be more numerous. To minimize the loss of true biologic haplotypes in population analyses, we have implemented an option in our population clustering to check if a cluster marked as possibly chimeric appears in another sample as one of the dominant haplotypes. If such a sample is found, the haplotype in question is, in that sample, unlikely to be chimeric since ideally a chimera would have two parents greater in abundance than itself. In this case, the putative chimera can be recovered in the original sample where it was at low-abundance. It is important to note that this step may not recover all true haplotypes as they might never appear at a high abundance in another sample. Also, a low-level chimera could be reinstated as a true haplotype. As there is no optimal solution for defining chimeras, we recommend every effort should be made during the PCR step to decrease likelihood of chimera formation. Also, chimera removal should be carefully considered and tuned, preferably with adequate controls, for the specific biology and experiment conditions. Again, these are options and during the sample and population clustering step it is possible to keep all putative chimeras for further analysis or to apply other chimera detection methods.

#### OTU clustering

SeekDeep also offers classical OTU clustering, which is slightly modified to be calculated by taking into account only errors not characterized as low k-mer frequency or low quality mismatches and optionally weighing indels in homopolymers less when analyzing 454 and Ion Torrent data. In this way, the percent identity calculated takes into account only likely biological differences between sequences.

See http://baileylab.umassmed.edu/SeekDeep/qluster_usage for full qluster usage information.

### processClusters: replicate and population comparisons

The qluster algorithm removes sequencing error and low level PCR error, but rare high-abundance errors due to polymerase errors in early rounds of PCR amplification are not easily discriminated. Therefore, when available, the SeekDeep pipeline uses PCR replicates (independent parallel amplifications of biologic sample aliquots) to identify and remove such errors – as early PCR errors should occur only in a single replicate, while biologic differences should occur faithfully in all samples. To compare replicates, the clustering results from each PCR are pooled and clustered again using the qluster algorithm. After this cross-replicate clustering, a replicate number cutoff is applied, which defaults to the number of replicates used; for example, if three replicates were analyzed, the default would require all three replicates contain a given haplotype. Though PCR replicates are recommended they are not required for SeekDeep to run.

Additionally, a cutoff for the fraction of total reads within the cluster can also be given for comparison; if the average fraction of a new cluster is not above the cutoff, the new cluster is removed. This cutoff defaults to 0.005 (0.5%), a generally conservative cutoff to minimize false haplotypes for the vast majority of experimental conditions, but can be set to more appropriate levels. For chimera filtering, if the majority of a cluster is made up of reads marked as possibly chimeric, it is also marked as chimeric and is removed by default. Final relative abundances for haplotypes are re-calculated after cutoffs have been applied and when replicates are available the final abundances of a haplotype is calculated by averaging the abundances across the replicates.

In addition to replicate processing and applying final cutoffs, processClusters can also assess the haplotypes across samples to provide population-level statistics. Once each sample has been processed, information is then collated across biologic samples within the defined population for each haplotype.

### popClusteringViewer: viewing and manipulating final results

A web server has been added to the SeekDeep suite to aid in the visualization and exploration of final results; this can be very helpful with large sample sets. The viewer is interactive and allows rapid exploration of final consensus sequences and the population haplotypes. It can also be used to extract subsets of the data. The viewer can easily be run on an individual's computer and can also be broadcast over the internet to provide persistent access to additional individuals.

See http://baileylab.umassmed.edu/SeekDeep/popClusteringViewer_usage for full usage information.

### Performance studies

To validate performance of the SeekDeep pipeline, we used two types of data. The first was simulated 454 and Illumina datasets. The second was actual PCR-amplified and sequenced (by Ion Torrent, 454 and Illumina) control mixtures of DNA from strains of several different pathogens to create mock mixed infections, which were collected from several previous studies and work in our own lab. We also used available mock bacterial communities. See below for a detailed description of these datasets.

#### Simulated datasets

The 454 and Illumina simulated datasets were created to test theoretical limits of detection for SeekDeep and other popular programs. The 454 datasets were simulated with 454sim (10) and Illumina datasets were created with ART (11). While a specific Ion Torrent simulator could not be found, the 454 simulator should provide results representative of Ion Torrent pyrosequencing given their similarities. An in-house program was used to generate the PCR error by simulating the rounds of PCR where a PCR error that occurred in an earlier round would appear at higher abundance than latter round errors, a feature not available in other PCR simulators. The program takes a starting DNA template amount, PCR error rate, a fasta file with relative abundances for reference haplotypes to simulate, and the number of rounds to simulate. For these simulations we used 2000 copies of starting DNA template, a PCR error rate of 3.5e–6 (representative of high-fidelity polymerases) and 30 rounds of PCR. Given the complexity of their formation, chimeras were not simulated.

Two mock haplotype mixtures were simulated to generate multiple test conditions:

- **Mock haplotype mixture 1 (minor versus major):** This mixture tests the ability of programs to discriminate minor haplotypes at various levels of divergence and abundance from a major abundant haplotype (Supplementary Figure S4A); thereby assessing the likelihood of minor haplotypes being collapsed into the major as probable error. Specifically, we simulated seven different haplotypes with increasing base mismatches (decreasing % identity) of 1 (99.7%), 2 (99.4%), 3 (99.1%), 4 (98.8%), 6 (98.2%), 8 (97.6%) and 13 (96.1%) from the major haplotype, with no shared mismatches between minor haplotypes to create distances always greater to other minor haplotypes than to the major haplotype, e.g. the minor haplotype with one mismatch and the minor haplotype with 2 mismatches from the major haplotype are three mismatches away from each other. The relative abundance of the minor haplotypes were simulated at 10%, 5%, 2%, 1%, 0.5%, 0.25%, 0.1% and 0.05% (Supplementary Figure S4B).
- **Mock haplotype mixture 2 (minor versus minor pairs with varying differences)**: This mixture examined the effect of divergence between minor haplotype pairs unrelated to the major haplotype (Supplementary Figure S4C). For this, we simulated 15 different haplotypes, making one major abundant haplotype and 14 minor haplotypes. Each minor haplotype was paired with another closely related minor haplotype, and each haplotype in the pair differed by at least 15 mismatches from the other pairs or to the major haplotype. Pairs had a range of base mismatches (% identity) consisting of 1 (99.7%), 2 (99.4%), 3 (99.1%), 4 (98.8%), 6 (98.2%), 8 (97.6%) and 13 (96.1%) nucleotides. The relative abundances of the minor haplotypes were simulated at 5%, 2%, 1%, 0.5%, 0.25%, 0.1% and 0.05% with the rest composed of the major haplotype. (Supplementary Figure S4D).

For each mixture and minor haplotype abundance above, we generated simulated datasets with two replicate PCRs with 2,000–10,000 reads incrementing by 2000 and at 50 000 reads (to test the extremes of coverage) for a total of six different read depths (equivalent throughout to nonredundant read or stitched-read coverage across the amplicon—or equivalently per base). Each of these conditions was simulated 10 times and the results were averaged to get the best estimate of program performance.

#### Known control mixture datasets

Five different experimental *in vitro* control mixtures were analyzed spanning the common sequencing technologies; 454, Ion Torrent and Illumina (Table 1). This included data from a eukaryotic parasite (*Plasmodium falciparum*) and a mock microbiome. Specifically these were:

- ***Plasmodium falciparum* control mixtures, 454 and Ion Torrent:***Plasmodium falciparum* control mixtures from our labs were sequenced on Ion Torrent and 454 (Table 1). These pools contained three different amplicons: thrombospondin-related anonymous protein (*TRAP*) (Supplementary Figure S5), apical membrane antigen 1 (*AMA1*) (Supplementary Figure S6), and circumsporozoite protein (*CSP*) (Supplementary Figure S7). The *AMA1* and *TRAP* samples had the same mixture of five strains: 40% K1, 30% 7G8, 15% Dd2, 10% RO33 and 5% V1/S and the *CSP* region had a mixture of 40% K1, 30% 7G8, 20% DD2 and 10% RO33 (Supplementary Figures S5 and S7).
- ***Plasmodium falciparum* control mixtures, Illumina MiSeq**: Additionally, twenty-eight different regions, including vaccine candidates and drug resistance genes, were PCR amplified and sequenced with 2 × 250 paired-end Illumina MiSeq from a control mixture of *P. falciparum* (Table 1). The mixture consisted of the following strains and relative abundances; 3D7 (∼79%), HB3 (∼7%), 7G8 (∼7%) and DD2 (∼7%). These targets included multiple probes in important vaccine candidate regions in *AMA1, CSP* and merozoite surface protein 1 (*MSP1*). Also known drug resistance or associated loci were targeted including apicoplast ribosomal protein S10 (*ARPS10*), multidrug resistance protein 1 (*MDR1*), multidrug resistance protein 2 (*MDR2*), kelch13 (*K13*), protein phosphatase (*PPH*), cytochrome *b* (*CYTB*), dihydrofolate reductase thymidylate synthase (*DHFR-TS*), and dihydropteroate synthase (*DHPS*) (Supplementary Figure S8).
- **Mock Microbiome:** Previous mock microbiome datasets by Salipante *et al.* were analyzed consisting of Illumina paired-end sequencing of the V1 region of the 16S coding region with three PCR replicates (24). This mock microbiome mixture contains 20 species, but due to highly similar copies within each species the number of expected haplotypes at one-base resolution for the V1 region is 47. Twenty of these haplotypes are only one base pair different from another haplotype. The 3 PCR replicates were deeply sequenced with approximately 800 000 reads each. To analyze data at more commonly assessed read depths (3,4,25) the replicates were downsampled to depths between 2000 and 20 000 increasing by intervals of 2000. Each read depth was sampled 10 times each for all three PCR replicates which generated a total of 300 different randomly sampled datasets.
- **Epstein-Barr Virus (EBV) and Human immunodeficiency virus type 1 (HIV) controls**: To provide a broader set of biologic examples, we also examined available viral controls of amplicon sequencing consisting of a previous mock HIV mixture (26) and a mock EBV mixture from our lab. The HIV dataset had five strains mixed together; 89.6 (10%), HXB2 (14%), YU2 (16%), NL4-3 (24%) and JR-CSF (36%). The mixture was sequenced five different times, two of the replicates were chosen and due to the great depth (>600 000) were each downsampled to 10 000 reads 10 times each for a total of 20 randomly sampled datasets. The EBV dataset was mixtures of an EBV type 1 strain and an EBV type 2 strain with frequencies ranging from 1% to 90% and a monoclonal sample of the type 1 strain. See Table 1 for more details.

**Table 1.** *In vitro* control datasets

Dataset amplicon	Technology	Read depth^a^	Sample number	Replicate^b^	Read length	Region length	Unique haplotype number	Range of haplotype base differences (% identity)^c^
PfTRAP	454	812–987	1	2x	345	345	5	1 (99.7%)–7 (97.9%)
PfAMA1	Ion Torrent	1323–1712	2	2x	494	494	5	2 (99.1%)–12 (94.9%)
PfCSP	Ion Torrent	1054–6403	4	2x	319	319	4	2 (99.3%)–9 (97.2%)
Various *P. falciparum* targets^d^	Illumina MiSeq	614–4497	28	None	2 × 250	330–403	2–4	1 (99.7%)–17 (95.3%)
Microbiome 16S-V1	Illumina MiSeq	584 575–899 804	1	3x	2 × 250	280	47	1 (99.6%)–101 (63.9%)
EBV	Illumina MiSeq	342–1350	6	2x	2 × 250	372	2	20 (92.6%)
HIV	Illumina MiSeq	10 000	20	2x	2 × 250	206	5	2 (99%)–5 (97.5%)

^a^Read depth equals number of stitched read pairs with minimum and maximum observed depths in the case of multiple samples and replicates.

^b^2x = two independent PCRs; 3x = three independent PCRs, or none = no replicate (single PCR) done.

^c^Number of differences are enumerated and followed by the corresponding percent identity, the range is shown when there are more than 2 unique haplotypes.

^d^Summary of the 28 targets here, see Supplementary Table S1 for details for each target.

MED (version 2.1), DADA2 (version 1.0.3), UNOISE (USEARCH version 9.2), and SeekDeep (version 2.4.0) were each run on the datasets with default or recommended parameters (See **Additional file 2** for details). The program ShoRAH (18) (version 1.1.0) was used on the viral datasets to represent a standard program for viral analysis. DADA2 and UNOISE have their own chimera detection program; MED and ShoRAH do not have a chimera-detection utility, so our own chimera detection was applied to the final results produced by MED and ShoRAH to make the results comparable. Each program has a different output format from which the consensus sequences and relative abundances of final clusters were extracted. The expected abundance of pooled species for each dataset was determined by aligning raw reads to reference sequences for that dataset. This calculation was performed because mock mixtures are manually produced in the lab, making the targeted mixture frequencies approximate. Common sources of experimental error arise by pipetting inaccuracy and imperfect amplification of the initial low abundance template leading to the introduction of random noise during the early rounds of PCR. Final clustering results were compared to the expected reference sequences and to determine which references were identified. For the paired-end Illumina data, sequences were stitched together with the program FLASH v1.2.11 (27).

To evaluate performance of each program we determined the number of expected haplotypes recovered—especially one-off haplotypes - and how well their abundances were predicted. We also determined the number and abundances of false haplotypes created. Recovery was calculated as the number of haplotypes exactly matching expected haplotypes divided by the total number of haplotypes expected. The haplotype recovery for MED, DADA2, and UNOISE was calculated based on each replicate separately, while SeekDeep's haplotype recovery was calculated if it found the expected haplotype in both replicates for a sample, as this is its default. Thus, SeekDeep's haplotype recovery is conservative relative to the other programs given that a haplotype must be present in both replicates to be counted as recovered.

All analyses and program comparisons were run on an Ubuntu 14.04 server with 64 2.4-GHz AMD processor cores and 512 gigabytes (GB) of RAM to allow parallelization of all simulations and *in vitro* datasets. For SeekDeep, all analyses presented could also be run individually on a laptop, a Macbook Pro with 16GB of RAM and a 4-core 2.4 GHz Intel i7 processor.

## RESULTS

### Simulation studies

**Figure 2.**
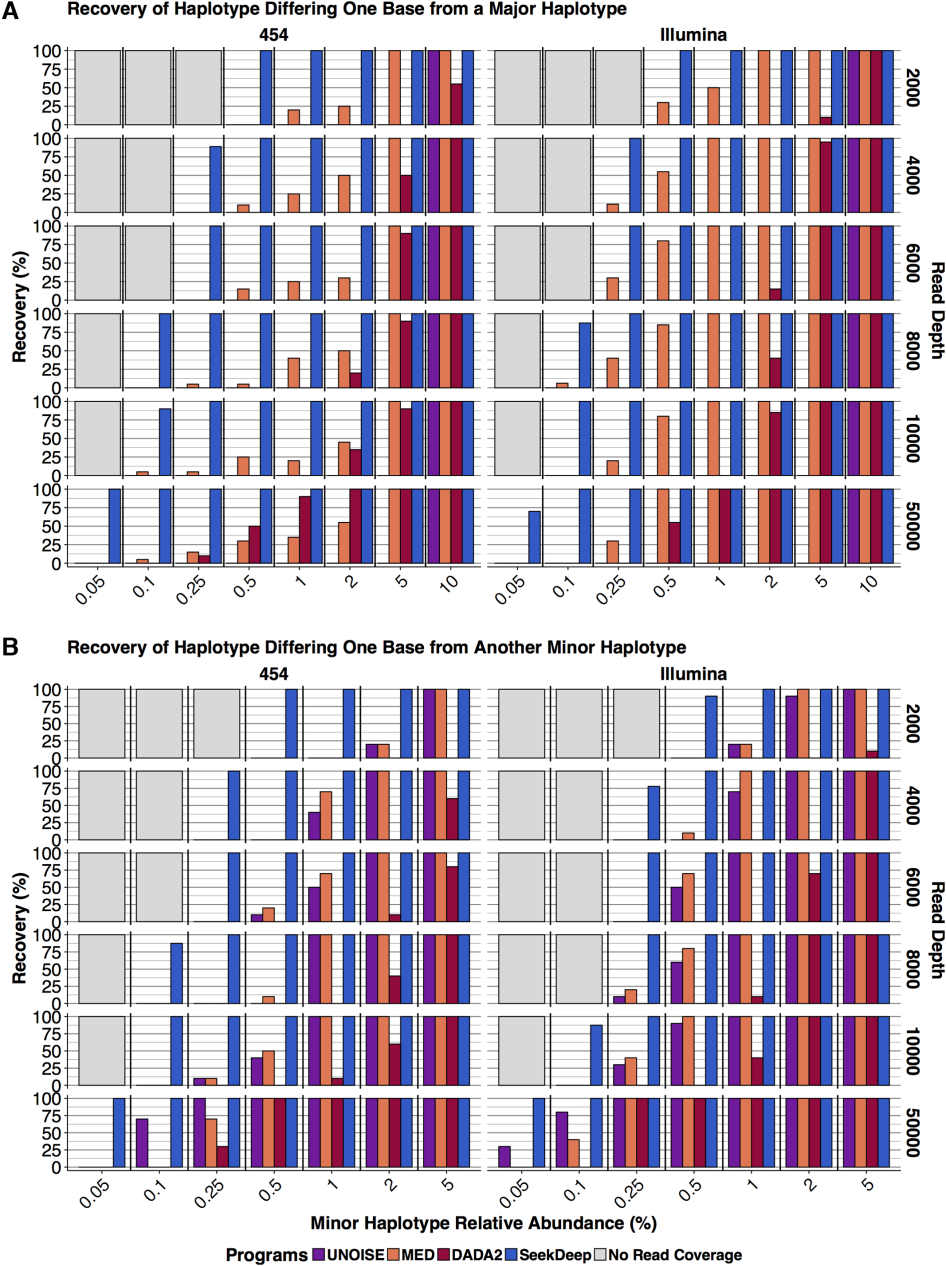
Haplotype recovery of simulated minor haplotypes differing by a single base. (**A**) Recovery of the haplotype differing by a single-base from a major haplotype in the mixture described by Supplementary Figure S4A and B. (**B**) Recovery of the two minor haplotypes that are one-off from each other described in the mixture described by Supplementary Figure S4C and D. For both panels, the y-axis represents the percent of simulations in which the haplotype differing by a single-base was detected and the x-axis represents the simulated expected abundance of the minor haplotype. Data is broken down by read depth (rows) and sequencing technology (columns), and bars are colored by program. Grey boxes at low-abundances represent combinations where the depth is not sufficient for reads to be observed for the minor haplotypes. For each minor haplotype abundance, there are 20 simulations from which DADA2, MED and UNOISE haplotype recovery was calculated as a percent of simulations in which the minor haplotype was detected. To best emulate real world situations in which a user would use SeekDeep to analyze replicates, we used paired simulations with the requirement that SeekDeep detect haplotypes in both simulations.

First we compared the performance of SeekDeep to the other programs on the two types of simulated mixtures: mixtures where minor haplotypes are closely related to a major haplotype which was at a much greater abundance (Supplementary Figure S4A and B), and mixtures where a minor haplotype was closely related to another minor haplotype at the same abundance (Supplementary Figure S4C and D). For all simulations, SeekDeep matched or outperformed MED, DADA2 and UNOISE in recovery of all haplotypes, especially one-off haplotypes (Figure 2). SeekDeep showed improved haplotype recovery compared to other methods, which was accentuated as read depth, divergence and abundance of haplotypes decreased (Figure 2, Supplementary Figures S9–S11). Together these factors combined to show marked differences in haplotype recovery for low-abundance haplotypes differing by a single-base (i.e. one off from the closest sequence) assessed with low numbers of reads (Figure 2). SeekDeep was also better able to estimate the expected abundance of haplotypes, demonstrated by a lower root mean squared error (RMSE) (Supplementary Figures S12 and 13) compared to all programs. The MED algorithm appears to have trouble as a haplotype's abundance increases, which could be due to the fact that it was developed specifically for microbiome data where the abundance of each haplotype usually does not exceed more than 10%. Though SeekDeep creates more false haplotypes than DADA2 and UNOISE, the abundance of the false haplotypes is generally much lower than 0.1% while DADA2, MED, and UNOISE were shown, especially for 454, to create false haplotypes greater than 1%, with most falling between 0.1% and 1% (Supplementary Figure S14). While DADA2 minimizes the number of false haplotypes (Supplementary Figure S14), it also loses sensitivity particularly with lower read depth input (Figure 2, Supplementary Figure S10). Overall, SeekDeep shows greater consistency at lower thresholds providing unbiased detection in the face of variable haplotype abundance and input read depths.

### *In vitro* control mixtures

#### Plasmodium 454 and Ion Torrent pyrosequencing

**Figure 3.**
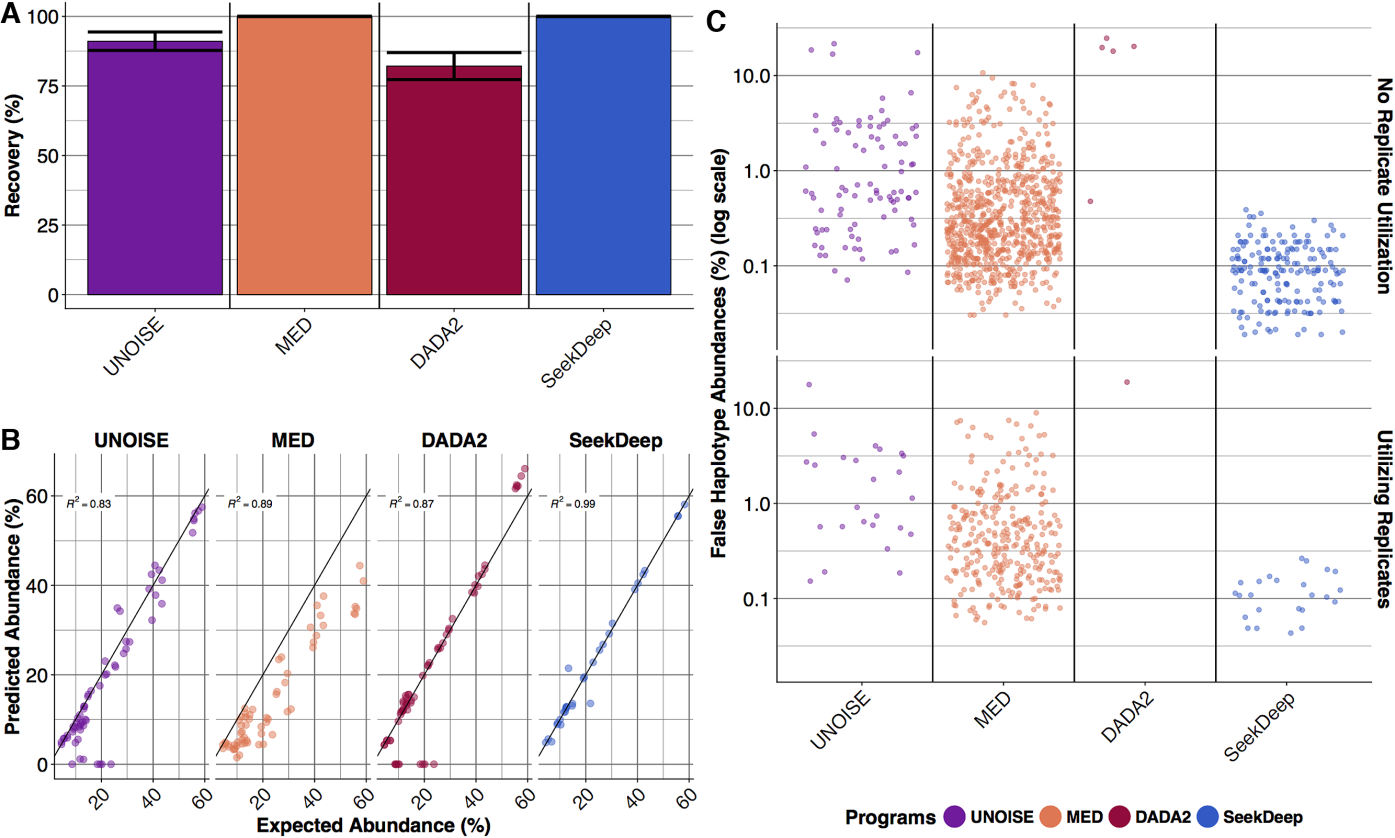
*In vitro* Ion Torrent and 454 mixtures performance. (**A**) The mean haplotype recovery for *in vitro* pyrosequencing samples with bars showing standard error. (**B**) Predicted abundance (y-axis) estimated by the various programs is plotted against the expected abundance (x-axis). Deviation from the line of identity represents the error and is summarized by the correlation coefficient. (**C**) False haplotypes are shown on a jitterplot to demonstrate their relative abundances and numbers (see Supplementary Table S3 for exact counts). Results are shown per program and also by the effect of utilizing or not utilizing replicates (haplotypes are only accepted if they appear in both replicates).

Next we evaluated the performance of haplotype detection for *P. falciparum* lab strains for *TRAP, AMA1* and *CSP* genes on both 454 and Ion Torrent by creating mock mixtures in the lab that were PCR amplified and then sequenced. This provides important insight into factors that may not be captured in the simulated sequence. For these *in vitro* mixtures, both SeekDeep and MED were able to achieve 100% haplotype recovery across all samples while UNOISE had 92% and DADA2 had 83% haplotype recovery (Figure 3A). Missed haplotypes usually represented the collapse of low-abundance highly similar haplotypes. In part, it also appeared that haplotype recovery for UNOISE and DADA2 were hampered by indel errors especially in homopolymers which are difficult to overcome in Ion Torrent and 454 data. This is a known issue as UNOISE’s website states that UNOISE does not work well on Ion Torrent and 454 data (http://www.drive5.com/usearch/manual/faq_unoise_not_illumina.html). All programs had appreciable false haplotypes (Figure 3C), and, while DADA2 had the lowest number of false haplotypes, when they did occur they often had appreciable frequencies even exceeding 10%. Only SeekDeep limited the occurrence of false haplotypes to low abundances (≤0.5%). Replicates again aided all programs but dramatically reduced the number of false haplotypes for SeekDeep. SeekDeep again showed the most accurate abundance estimates (Figure 3B). Notably MED, while demonstrating 100% haplotype recovery, consistently underestimated abundances due to the numerous false haplotypes at appreciable frequencies (Figure 3C).

#### Plasmodium Illumina MiSeq

**Figure 4.**
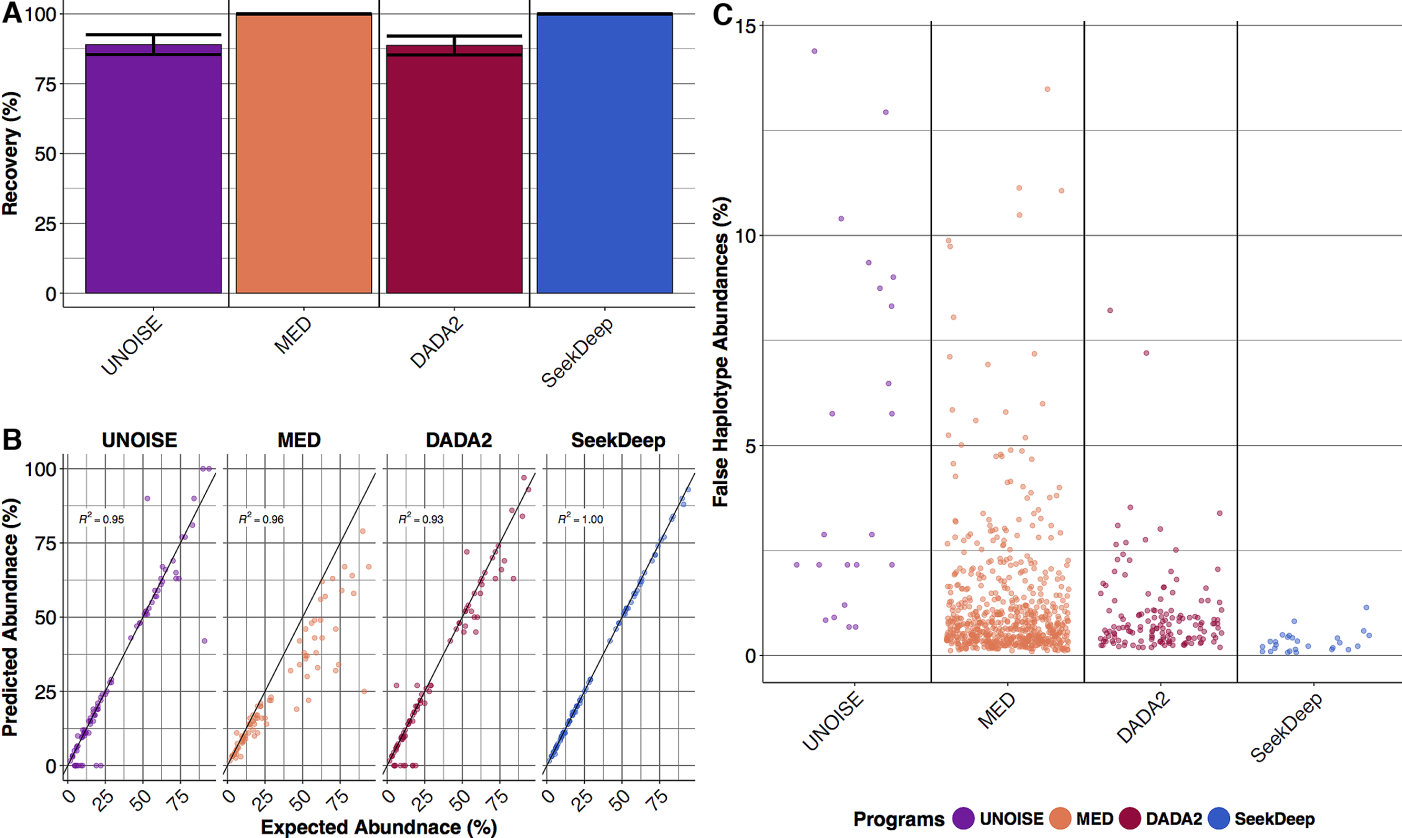
*In vitro* Illumina *P. falciparum* performance. (**A**) The mean haplotype recovery for *P. falciparum in vitro* Illumina datasets with bars showing standard error. (**B**) Predicted abundance (y-axis) estimated by the various programs plotted against the expected abundance (x-axis). Deviation from the line of identity represents the error and is summarized by the correlation coefficient. (**C**) False haplotypes are shown on a jitterplot to demonstrate their relative abundances and numbers (see Supplementary Table S4 for exact counts). No replicates were available for this dataset.

We also evaluated a mock mixture of *P. falciparum* across 23 loci that represent important markers of drug resistant or regions of diverse variation. These amplicons were PCR amplified and sequenced on Illumina MiSeq 2 × 250 paired end. SeekDeep and MED were able to achieve 100% haplotype recovery of all 23 targets while DADA2 and UNOISE both failed to detect nine out of the 88 total haplotypes. Five haplotypes were missed in common by both programs (Figure 4 and Supplementary Figure S15). The haplotypes that UNOISE and DADA2 failed to detect where either related to another haplotype by a single nucleotide or 1 large indel (∼10 nucleotides) and ranged in abundance from 4 to 20%. SeekDeep demonstrated a minimal number of false haplotypes on par with UNOISE (Figure 4C). Unlike UNOISE and the other programs which report false haplotypes at abundances that can exceed 10%, SeekDeep's false haplotypes were all less than 0.8% abundance. Again SeekDeep showed the highest accuracy in terms of predicting the abundance (Figure 4B).

#### Mock microbiome

We also tested SeekDeep on a mock microbiome dataset previously described in Salipante *et al.* (24), which had been amplified and sequenced in triplicate on the Illumina platform. It contained 47 distinct 16S copies (Supplementary Table S5). MED and SeekDeep were able to recover 100% of all expected haplotypes in all datasets, while DADA2 missed one haplotype. For all three replicates of this dataset, DADA2 missed the *L. monocytogenes*.2 haplotype, which had an expected abundance of 0.8% and is one nucleotide different from the *L. monocytogenes*.5 haplotype which had an expected abundance of 1.5%. UNOISE also missed *L. monocytogenes*.2 in one replicate and in all three replicates missed *B. vulgatus.3* (0.035%), *B. cereus.4* (0.33%) and *B. cereus.1* (0.36%), haplotypes, which all differ by one nucleotide from another haplotype.

#### Downsampled mock microbiome

Because the mock microbiome dataset previously described in Salipante *et al.* (24) was sequenced to a great depth (>600 000 reads), we randomly downsampled the dataset to lower read depths (2000–20 000) to test detection at levels of sequencing more commonly employed in experiments. For the downsampled mock microbiome dataset from Salipante *et al.* (24), SeekDeep outperformed DADA2, MED, and UNOISE in haplotype recovery of the twenty-three one-off haplotypes (out of 47 total haplotypes in the dataset). The highest relative abundance of missed one-off haplotypes was ∼3% for DADA2 and MED, 2% for UNOISE, but only 0.25% for SeekDeep (Supplementary Figure S16). Again, SeekDeep does well using fewer input reads in estimating the expected abundance of the known haplotypes with a lower RMSE (Supplementary Figure S17).

#### Viral strain mixtures

To further ensure that SeekDeep works across a breadth of experiments and organisms, we examined control mixtures of viral strains. All programs performed well with respect to recall for both the EBV (Supplementary Figure S18) and HIV (Supplementary Figure S19) mixtures, which was not unexpected as these mixed strain haplotypes all differed by more than a single base. Importantly, SeekDeep's specificity compared well. All other programs other than SeekDeep created false haplotypes >1% in the EBV dataset with the highest for each program being 2% for UNOISE, 17% for MED, 22% for DADA2, 19% for ShoRAH and 0.65% for SeekDeep which was mitigate but not completely removed with replicates (1% for UNOISE, 16% for MED,14% for DADA2, 16% for ShoRAH and 0.46% for SeekDeep). Programs performed better on the HIV dataset and though SeekDeep had a large number of false haplotypes all of them fell below the recommended cut off of 0.5% with the highest being at 0.35%. This high amount of apparent false haplotypes at low frequency was probably representative of both increased biologic variation due to HIV replication by error-prone reverse transcriptase as well as the elevated 65 rounds of PCR amplification prior to sequencing.

### Chimera detection

For these *in vitro* control mixtures, chimera formation and abundance was highly variable depending upon the experiment. The Illumina *P. falciparum* dataset only demonstrated 3 chimeras across all 28 amplicions. The IonTorrent controls demonstrated significant numbers of low abundance chimeras. Across the seven samples there were a total of 186 false haplotypes of which 83% (155) were chimeras. These IonTorrent false haplotypes generally showed higher abundances relative to other false haplotypes and were highly-reproducible abundances across replicates (*R*^2^ = 0.81–0.99; Supplementary Figure S20). The differences in chimera formation between datasets most likely originates from differences in the amount of input template and PCR conditions as well as potentially the library preparation which involves PCR. The mock microbiome showed minimal chimera formation likely due to the decreased sequence relatedness and greater amounts of starting template. Overall, the variability in chimera occurrence rates along with their high-degree of reproducibility within replicates emphasizes the need to carefully consider the experimental conditions and the utilization of experimental controls to determine the need and optimal settings for chimera detection.

### Traditional microbiome OTU analysis

In addition to providing single-base resolution between sequences, SeekDeep was designed to also allow users to define the needed level of resolution by setting either the number of bases or percent identity to create operational taxonomic units (OTUs). We therefore compared SeekDeep to older commonly used programs offering OTU level resolution that can operate on multiple platforms. In comparison to USEARCH (i.e. UCLUST), Seekdeep showed both better accuracy and precision clustering at 97% OTUs (Supplementary Figure S21). Also, USEARCH at times misconstructs the OTUs, returning a consensus sequence that is not one of the actual input haplotypes (Supplementary Figure S21B). SeekDeep routinely returns the major haplotype within an OTU. We also compared to SWARM collapsing on 1-base differences—the most sensitive setting for SWARM (Supplementary Figure S22). Again SeekDeep demonstrated better haplotype recovery and fewer false haplotypes. Thus, SeekDeep provides more optimized OTU definition, which again is more robust to varying read depth.

### Performance

Algorithm speed can be an important factor in terms of practicality, and SeekDeep compares favorably with other programs. While UNOISE is the fastest algorithm (Supplementary Figure S23), this speed comes at a cost (Figure 2). The proprietary algorithm in UNOISE works by collapsing one-off errors if the ratio of abundance between two sequences is at a certain threshold, which precludes UNOISE from detecting new haplotypes that differ by only one nucleotide from the major haplotype in the population. This aspect can be problematic when screening for cancer mutations or pathogen drug resistance. Also UNOISE recommends not using singlet sequences, decreasing haplotype recovery at lower read depths. This, in part, contributes to its speed (Supplementary Figure S23) but decreases haplotype recovery. Apart from UNOISE, SeekDeep is comparable in speed to DADA2 and MED (Supplementary Figure S23). In fact, for the mock microbiome data set (24), which had ∼800 000 for each of three replicates, the run times for the programs were 2 h and 41 min for SeekDeep, 2 h and 40 min for DADA2 and 1 h and 58 min for MED on standard hardware as found in a personal computer. Given runtimes are comparable, the built-in general pipelines for sample processing make SeekDeep a potentially less-time consuming option for the general user looking to process numerous samples and multiple amplicons per sample.

## DISCUSSION

With newer sequencing technologies increasing our ability to probe a wide variety of biologic samples, the ability to bioinformatically discern the full extent of sequence diversity, even if only a single-base difference, is key to answering many important questions. Though all programs tested are able to detect one-off haplotypes, SeekDeep is the only one consistently able detect these haplotypes at lower frequencies and at lower input read depths for all technologies (Figure 2, Supplementary Figures S9–S11, S16). SeekDeep performs well across a diverse set of simulations and *in vitro* control data sets and provides a more favorable balance between haplotype recovery and false haplotypes such that missed haplotypes and false calls are limited to the lowest frequencies, usually below 0.25%. In fact, when applying 0.25% as a lower threshold, SeekDeep has near perfect haplotype recovery and precision (Supplementary Figure S24). We apply a slightly higher cutoff of 0.5% as the default in processClusters, the final processing step, ensuring high confidence in the called haplotypes. Essentially, SeekDeep provides the ability to confidently detect haplotypes across variable read depths regardless of haplotype abundance, similarity or platform, a feature which is crucial for maximizing experimental information and minimizing biases. Minimizing bias is important for downstream analyses such as time series that generally presume random deviations (28,29).

SeekDeep showed important differences in terms of haplotype recovery and false haplotype creation compared to other programs. While DADA2 creates a smaller number of false haplotypes than SeekDeep, this comes at the cost of missing low-abundance one-off haplotypes. Also, when DADA2 does create a false haplotype it is generally at a higher abundance than SeekDeep (Figures 3 and 4, Supplementary Figure S14). DADA2 did not compare well at lower read depths where haplotype recovery suffered remarkably. Thus, for users of DADA2 it may be important to ensure that all samples have deep read depth to minimize biases. MED has good haplotype recovery but also creates numerous false haplotypes, particularly high-abundance haplotypes in samples with low diversity (Figures 3 and 4, Supplementary Figure S14). SeekDeep's balance between haplotype recovery and false haplotypes at very low-abundances was by design. For subsequent aggregate or longitudinal analyses across samples, low-level noise in individual samples can often be better controlled across the entire sample set. However, missing haplotypes or false calls at appreciable levels are more difficult to compensate for and can be a source of significant bias.

Importantly SeekDeep is extremely robust to sequence quality or types of sequence variation. SeekDeep directly utilizes the actual base quality of each sequencing read. Thus, it is robust to sequences that are outliers with extremely poor quality. Unlike both MED and DADA2 that require that input sequences be the same exact length, SeekDeep can handle variable length input given it performs optimal global alignments, and thus is adept at analyzing sequences with insertions or deletions. Variable length inputs are very common among Ion Torrent and 454 sequencing data.

Users can further optimize SeekDeep for more advanced applications. It can flexibly cluster based on insert size, allowing for the detection of biologically relevant insertions such as nucleotide triplets consistent with an amino acid change while filtering out homopolymer or smaller indels that are particularly common in some sequencing platforms such as IonTorrent. With SeekDeep, users can set the specific number of each type of alignment differences (indels and/or SNPs) upon which to collapse clusters, enabling concrete tuning for the specific biologic questions. For instance, this allows a user to collapse haplotypes that differ by one base, two bases, or traditional analyses collapsing to 97% or 99% OTUs and detect more divergent lower abundance variants that may be only represented by a few sequences in a sample.

SeekDeep offers robust and flexible pre- and post-clustering tools and workflows for rapidly preprocessing numerous samples by demultiplexing barcodes, identifying and removing primers, trimming, and cleaning sequence to user specifications. After clustering, the tool set helps evaluate the sequence and perform initial data evaluation with key sample and population statistics. SeekDeep has built-in support for a number of steps including (i) scanning for contamination, which is especially helpful, for example, in *Plasmodium* datasets which can often be contaminated with human DNA due to low relative amount of parasite DNA, (ii) built-in support for incorporating replicate comparison and (iii) support for analysis of multiple amplicon targets at once. It also supports chimera detection and removal akin to other programs which should be carefully considered and tuned based on experimental conditions, controls and the biologic question of interest. SeekDeep also provides a dynamically interactive HTML viewer, which makes it easy to explore differences between strains and has support for viewing results on subgroups in large sample sets when given group metadata.

Overall, SeekDeep expands the potential for *de novo* amplicon clustering—particularly given its improved haplotype recovery at lower read depths for haplotypes differing by one base. This is crucial for projects that seek to detect and quantify minority haplotypes that may be represented by a single SNP. Such projects are becoming increasingly common in the oncology and infectious disease fields. For example, when using marker regions to differentiate bacterial strains, or when monitoring for pathogen drug-resistance mutations, these sequences often only differ from the wild type by a single base (30). Accurately quantifying these low-abundance and genetically similar strains in these cases is key.

In summary, SeekDeep can be widely applied to all forms of amplicon deep sequencing to improve the haplotype recovery of highly-similar sequences while minimizing false haplotypes across a broad range of relative frequencies, read depths and platforms. This should allow users to maximize information extraction while minimizing biases in their downstream analyses and conclusions. In addition, the full SeekDeep suite of tools for pre- and post-processing will speed clustering optimization and provide high-quality and interpretable haplotype data for further analysis.

## AVAILABILITY

Source code for the current stable release of SeekDeep can be found at https://github.com/bailey-lab/SeekDeep and full usage and tutorials can be found at the SeekDeep website. For full install information see http://baileylab.umassmed.edu/SeekDeep/installingSeekDeep.

The *in vitro* data can be found via their original publications. The simulation raw data and the *P. falciparum* Illumina MiSeq data can be found at http://baileylab.umassmed.edu/data/SeekDeepPaperData.

## Supplementary Material

Supplementary Data
