# Supplementary material for: SeekDeep: single-base resolution de novo clustering for amplicon deep sequencing
Source: Nucleic Acids Res. 2017 Nov 30;46(4):e21. doi: 10.1093/nar/gkx1201 (PMC5829576; doi:10.1093/nar/gkx1201)

Figure S1 - PCR and Sequencing Errors
Figure S2 - Overview of the qluster Algorithm
Figure S3 - Characterizing Errors in Pairwise Comparisons within qluster
Figure S4 - Simulated Mixtures
Figure S5 - In vitro P. falciparum TRAP Strain Mixture
Figure S6 - In vitro P. falciparum AMA1 Strain Mixture
Figure S7 - In vitro P. falciparum CSP Strain Mixture
Figure S8 - In vitro P. falciparum Illumina Strain Mixtures
Figure S9 - Haplotype Recovery of Simulation Data - Platform
Figure S10 - Haplotype Recovery of Simulation Data - Read Depth
Figure S11 - Haplotype Recovery of Simulation Data - Minor Haplotype Abundance
Figure S12 - Predicted vs Expected Haplotype Abundances for Simulations
Figure S13 - Predicted vs Expected Haplotype Abundances for Simulations of Closely Related Haplotypes
Figure S14 - False Haplotype Abundances from Simulations
Figure S15 - In vitro P. falciparum Illumina Mixtures Performance
Figure S16 - Down-sampled Mock Microbiome Haplotype Recovery of Haplotypes Differing by One Base
Figure S17 - Down-sampled Mock Microbiome Predicted vs Expected Haplotype Abundances
Figure S18 - In Vitro EBV Illumina Performance
Figure S19 - In Vitro HIV Illumina Performance
Figure S20 - Chimera Detection
Figure S21 - OTU Clustering Performance on Simulation Data
Figure S22 - Collapsing on Single-base Differences Performance on Simulation Data
Figure S23 - Program Run Times
Figure S24 - Haplotype Recovery of Expected Haplotypes and Creation of False Haplotype above ≥0.25% on Simulated Datasets

Figure S1

PCR and Sequencing Errors

Clustering of amplicon sequencing must contend with errors that occur during PCR and sequencing. **a)** Early round PCR errors can be difficult to identify because these propagate in subsequent rounds and can reach a relatively-high abundance. While usually uncommon, the degree of such high-abundance errors is highly dependent on the number of initial target DNA copies in the PCR. Thus, experiments that utilize nested PCR to amplify low DNA concentration samples are particularly prone to high-abundance errors. Low-abundance errors that occur in later rounds of amplification may be numerous but are more easily identified and removed. **b)** Another common problem in PCR, particularly when co-amplifying highly-similar sequences, is the creation of chimeras, which are formed when a partial PCR product re-anneals to a similar template creating a hybrid product.

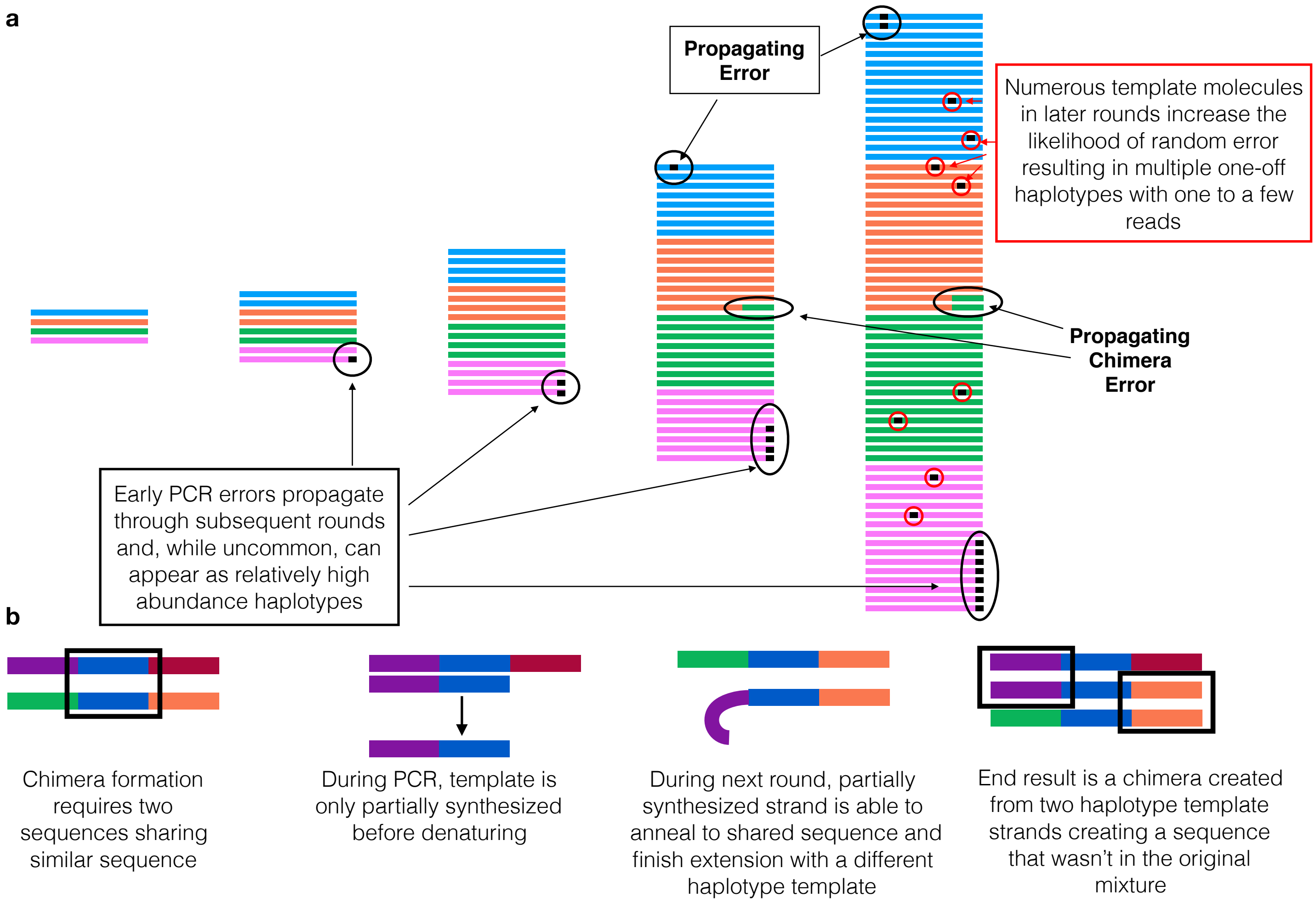

Figure S2

Overview of the qluster Algorithm

Qluster starts by operating on the initial reads that have already been demultiplexed by sample and primers. Qluster first **(1)** creates initial clusters of identical sequences and then sorts these clusters by read count in descending order. Pairwise global alignments are then used to compare the representative sequences and **(2)** collapse initially on a minimal amount of allowable errors (see **Figure S3** for depiction on how errors are characterized). Qluster thus collapses only the most similar sequences and creating larger aggregate clusters for further comparisons. After each collapse, a consensus sequence is created. On the next iteration comparing all clusters, the amount of **(3)** allowable error is increased to further collapse clusters. Further iterations **(4)** increase amount of error allowed, again creating a consensus after each collapse. Final clusters are created after the final iteration. After clustering, **(5)** mark any sequences that could be a possible chimera.

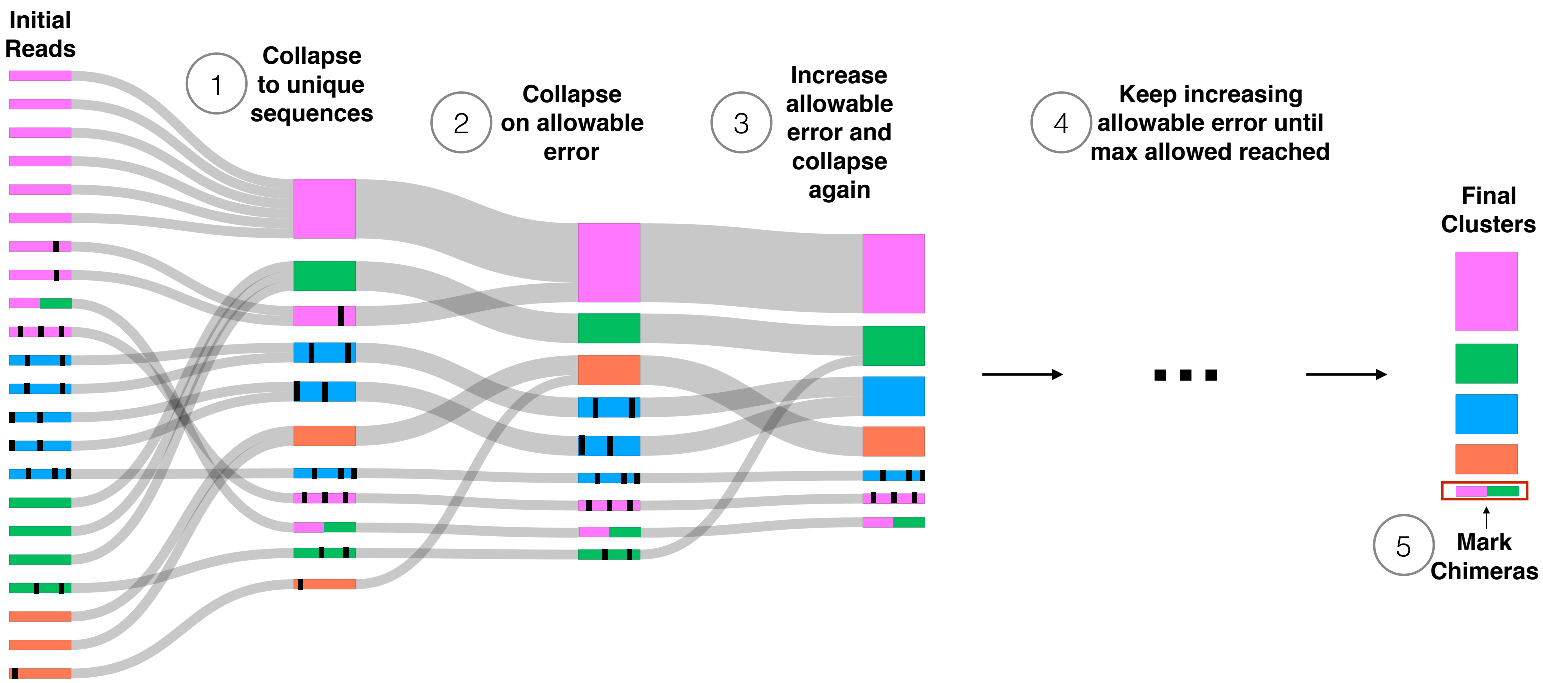

Figure S3

Characterizing Errors in Pairwise Comparisons within qluster

Clustering incorporates both base quality and abundance of k-mers as well as parameters relevant to the error profile of the sequencing platform. Depicted is a detailed example of how qluster scores and then determines whether to collapse two clusters. **a)** After pairwise global alignment of the cluster consensus, potential errors are categorized into indels and mismatches. **b)** Mismatches between sequences are first checked for base quality, which includes comparing the base quality scores of mismatching bases and the surrounding bases to a quality thresholds (default is 20 for mismatching bases and 15 for surrounding bases). Both the mismatch site and regional qualities must be higher than this threshold to be considered a high quality mismatch. **c)** High quality mismatches are then further classified by their occurrence in the input data based on the abundance of k-mers in each sequence centered on the mismatch. By default, if the k-mer only occurs once in the input data it is marked as a low abundance mismatch signifying likely error. **d)** Indels are classified by size and are classified into 1-base indels, 2-base indels, and > 2-base indels. Optional weighting for indels that occur in homopolymers can be turned on for pyrosequencing platforms (i.e. 454 and Ion Torrent). **e)** Errors are tabulated and then compared to the current thresholds to determine if the two given clusters should be merged or maintained. In the depicted example, the clusters are not merged as the number of high quality mismatches observed exceeds the threshold for collapse.

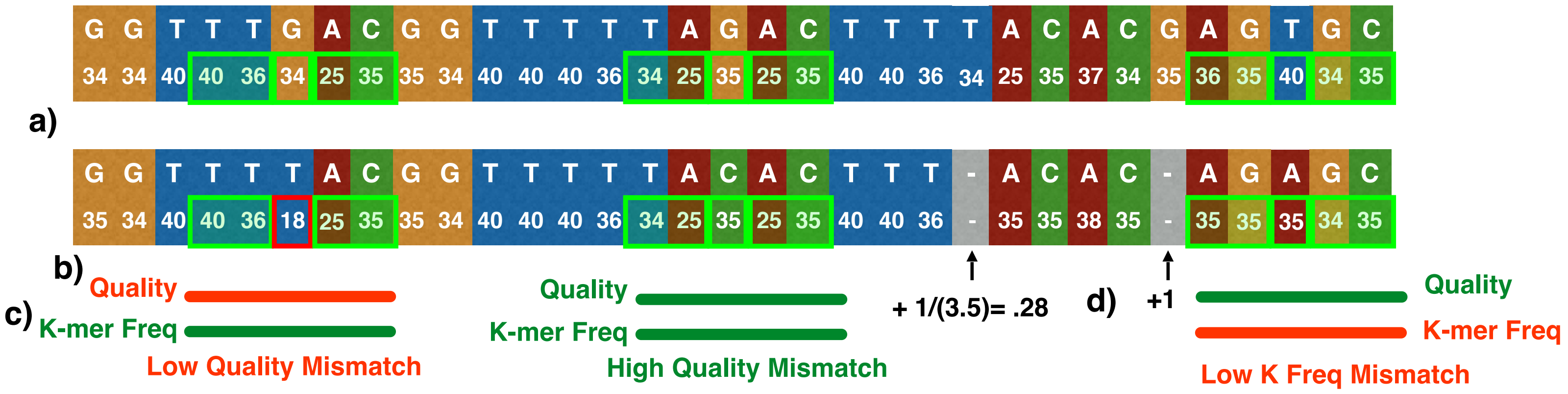

e)

Differences	*Allowable*	*Observed*
1 base indel:	2	1.28
2 base indel:	0	0
>2 base indel:	0	0
High Qual Mismatches:	0	1
Low Qual Mismatches:	1	1
Low K-mer Mismatches:	1	1

→ Exceeds **allowable** don't collapse

Figure S4

Simulated Mixtures

Two types of haplotype mixtures were simulated to assess performance. **a)** The first mixture tests discrimination of related low-abundant minor haplotype and highly-abundant major haplotype and is comprised of 7 minor haplotypes differing from the major haplotype by 1 to 13 differences. **c)** The second simulated mixture tests the ability to discriminate highly similar low-abundance haplotypes from each other. There are seven minor haplotype pairs differing by 1, 2, 3, 4, 6, 8, or 13 nucleotides. Between pairs and between the major haplotypes there are at least 15 nucleotides (all red dots not shown). **b)** The 8 different abundances at which haplotypes in panel **a)** were simulated. **d)** The 7 different abundances at which haplotypes in panel **c)** were simulated. Each was simulated 10 times at a variety of read depths. The number of red nodes between haplotypes is the number of base pair mismatches (bpm) differentiating them.

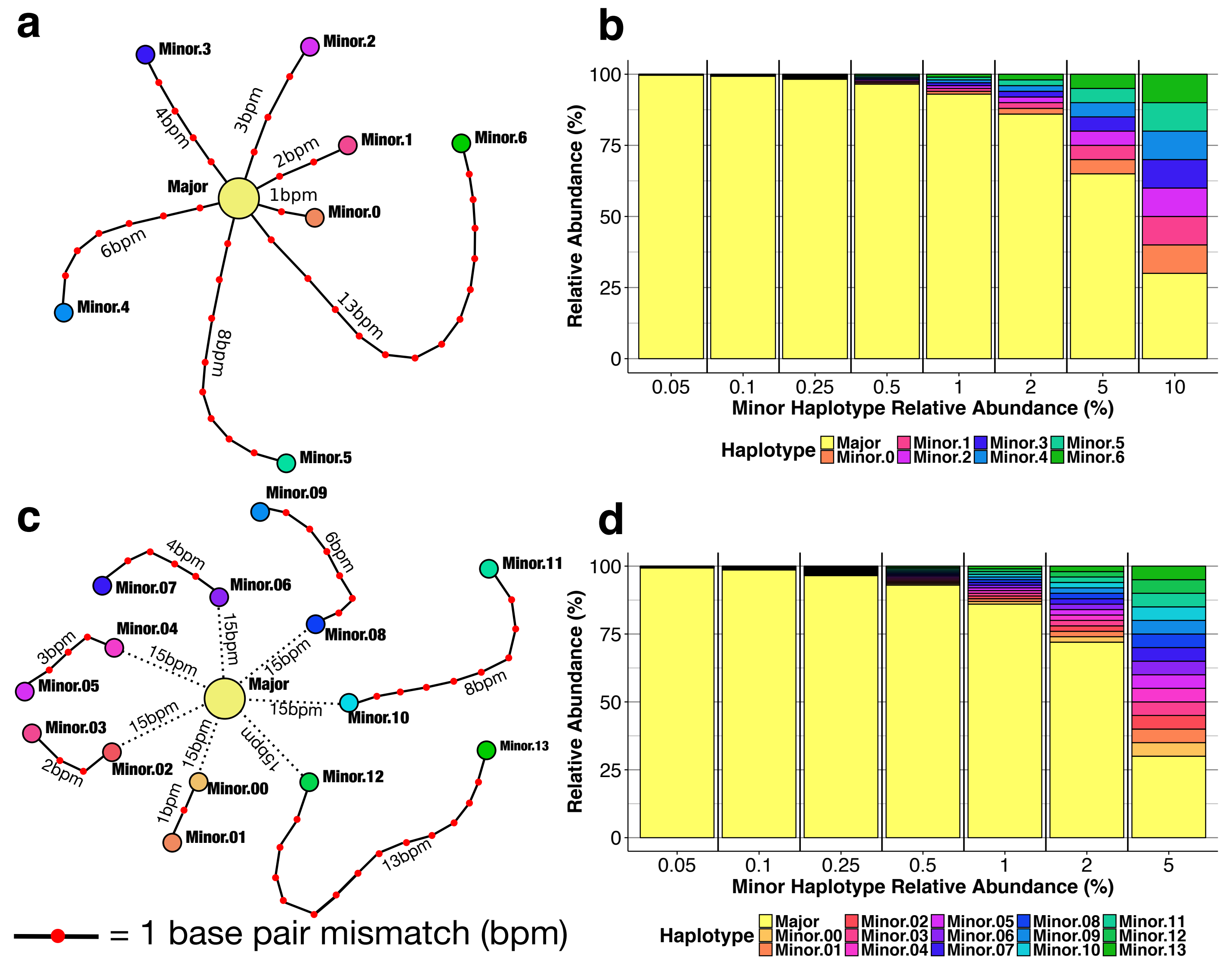

Figure S5

***In vitro P. falciparum TRAP* Strain Mixture**

The *TRAP* mixture consisted of 5 different *P. falciparum* strains. The mixture was amplified and sequenced twice. Panel **a)** gives the expected relative abundances for the mixture and **b)** is a distance matrix describing the number of base mismatches and percent identity between the strains.

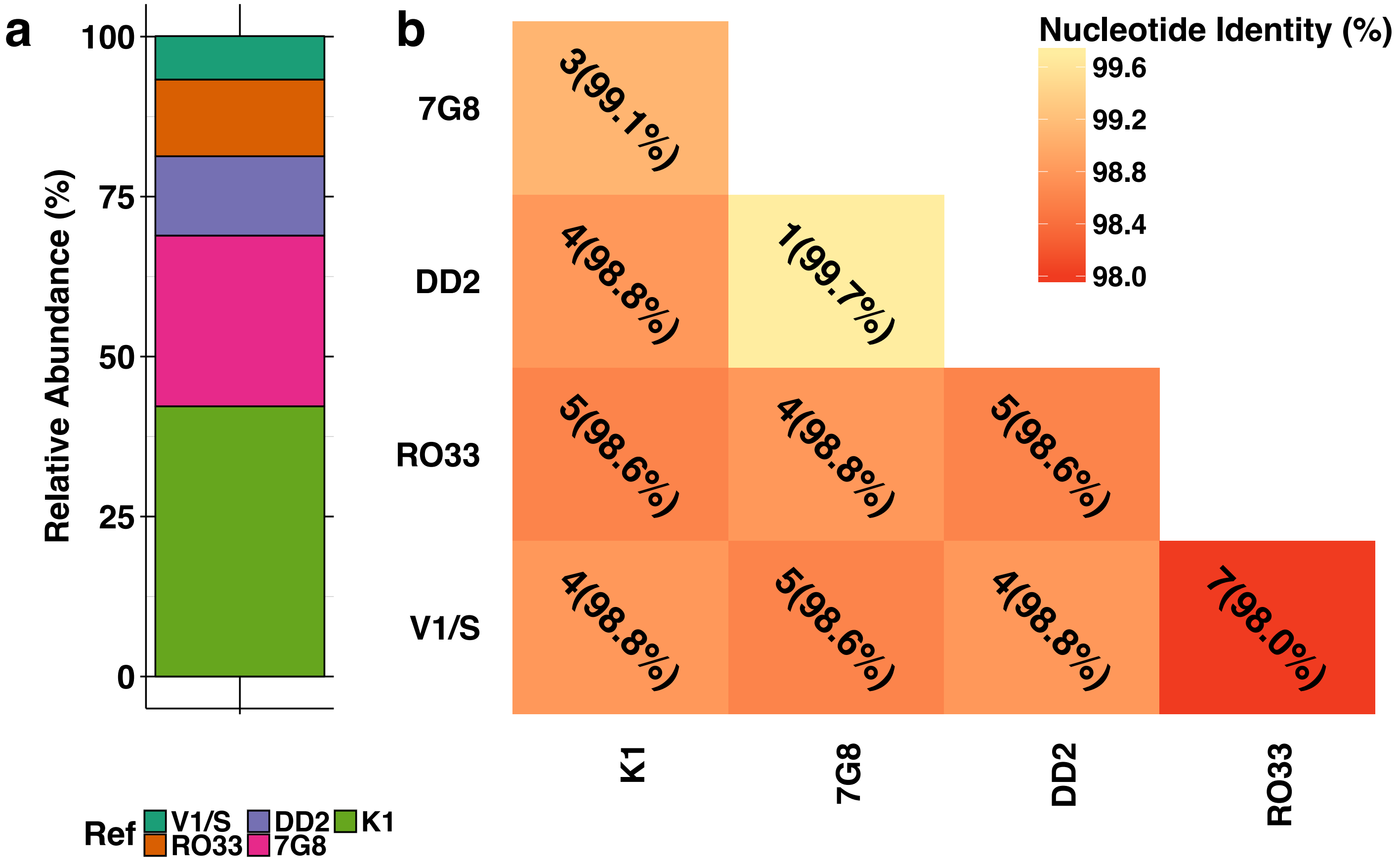

Figure S6

***In vitro P. falciparum AMA1* Strain Mixture**

The *AMA1* mixture consisted of 5 different *P. falciparum* strains. The mixture was amplified and sequenced 4 times. Panel **a)** gives the expected relative abundances for the mixture and **b)** is a distance matrix describing the number of base mismatches between the strains and the corresponding percent identity.

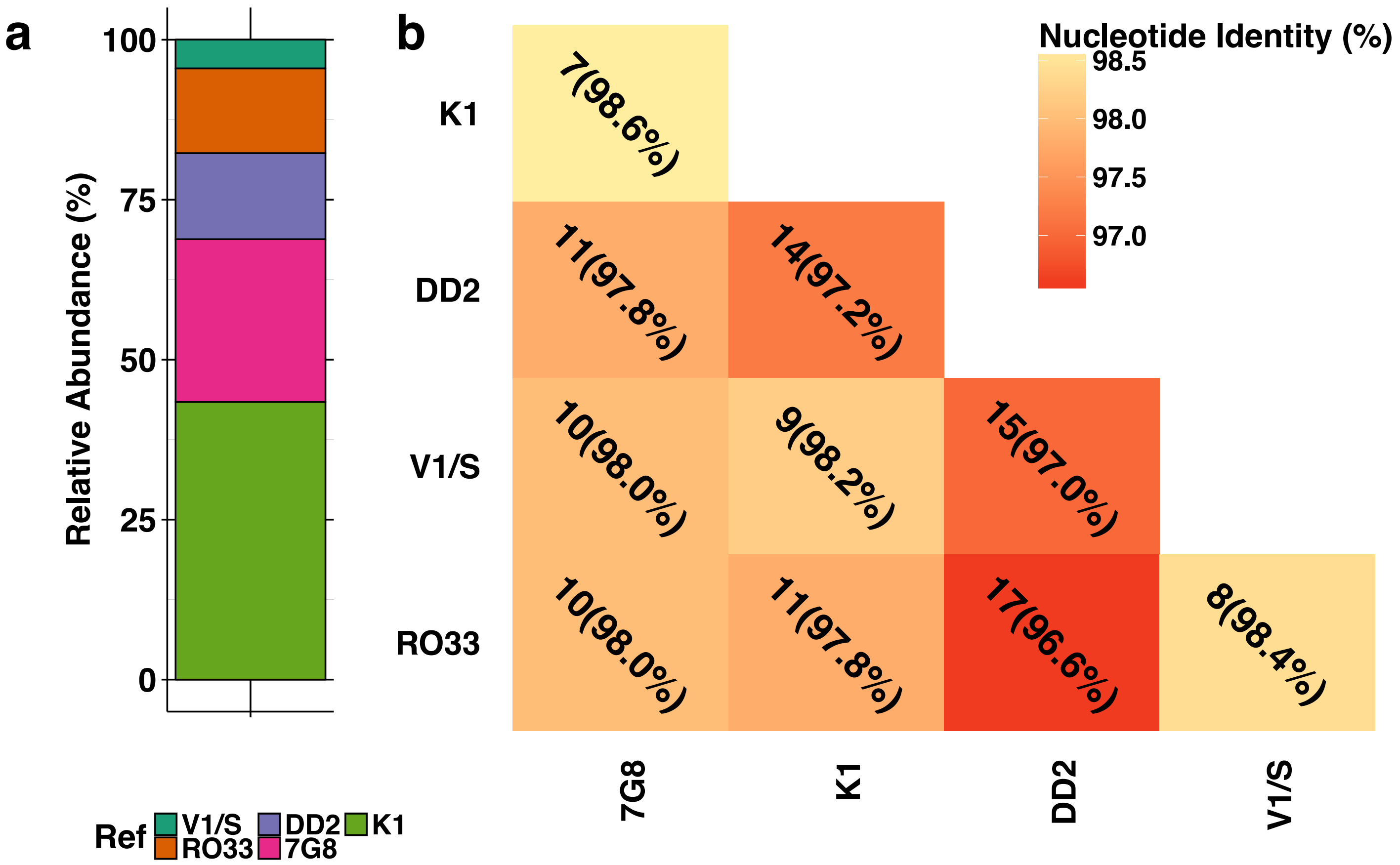

Figure S7

***In vitro P. falciparum CSP* Strain Mixture**

The *CSP* mixture consisted of 4 *P. falciparum* strains. The mixture was amplified and sequenced 8 times. Panel **a)** gives the expected relative abundances for the mixture and panel **b)** is a distance matrix describing the number of base mismatches between the strains and the corresponding percent identity.

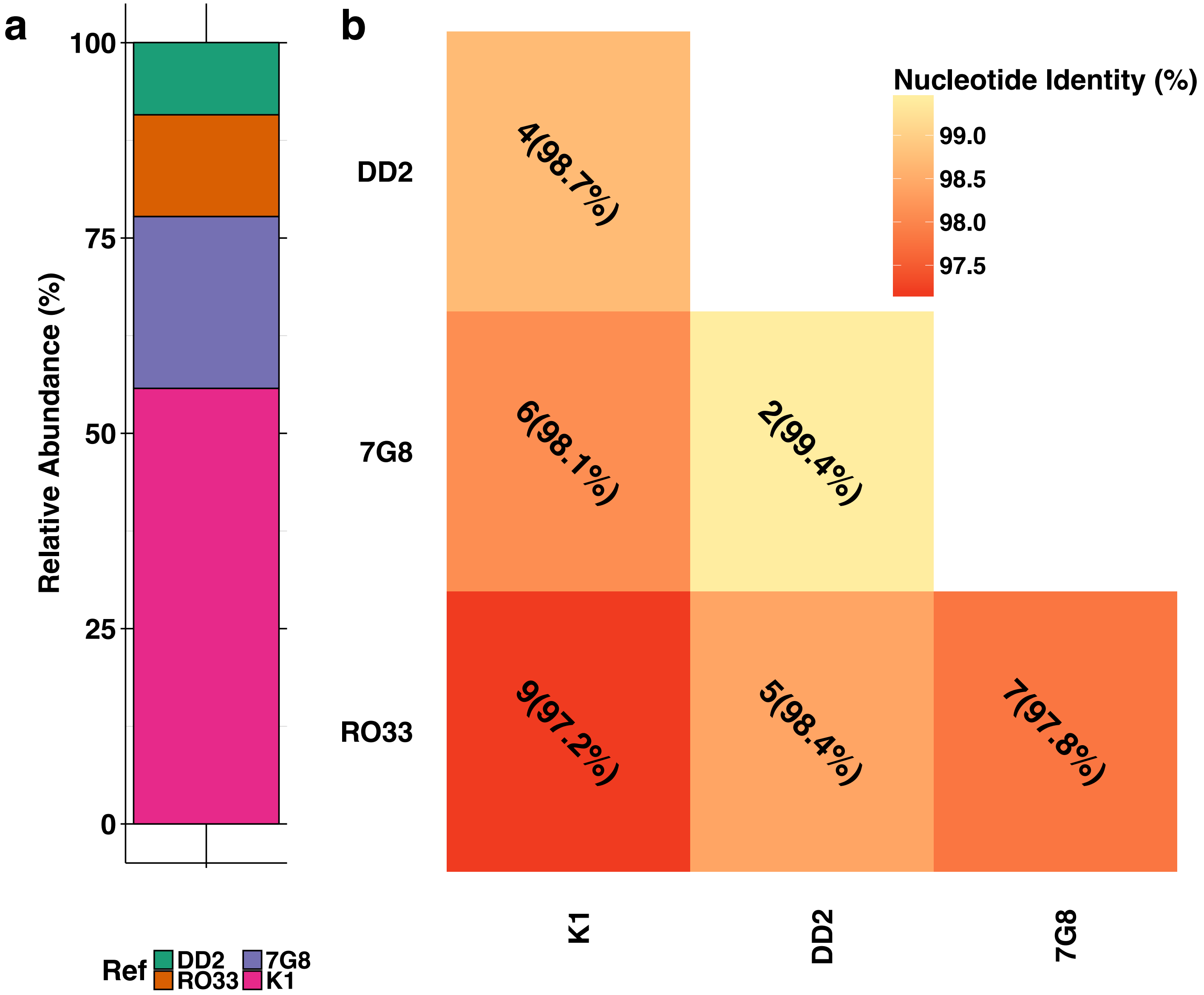

Figure S8

***In vitro P. falciparum* Illumina Strain Mixtures**

The expected abundances for all amplicons in the control mixture of the strains 3D7, 7G8, HB3, and DD2. While the mixture of the individual strains was constant (3D7 = 79%, 7G8 = HB3 = DD2 = 7%) for all amplicons the strains often shared the same haplotype leading to variation in the number and abundance of haplotypes (2-4) across the amplicons. Differences between strains range from 1-2 SNPs and sometimes large indels (10-15 bp).

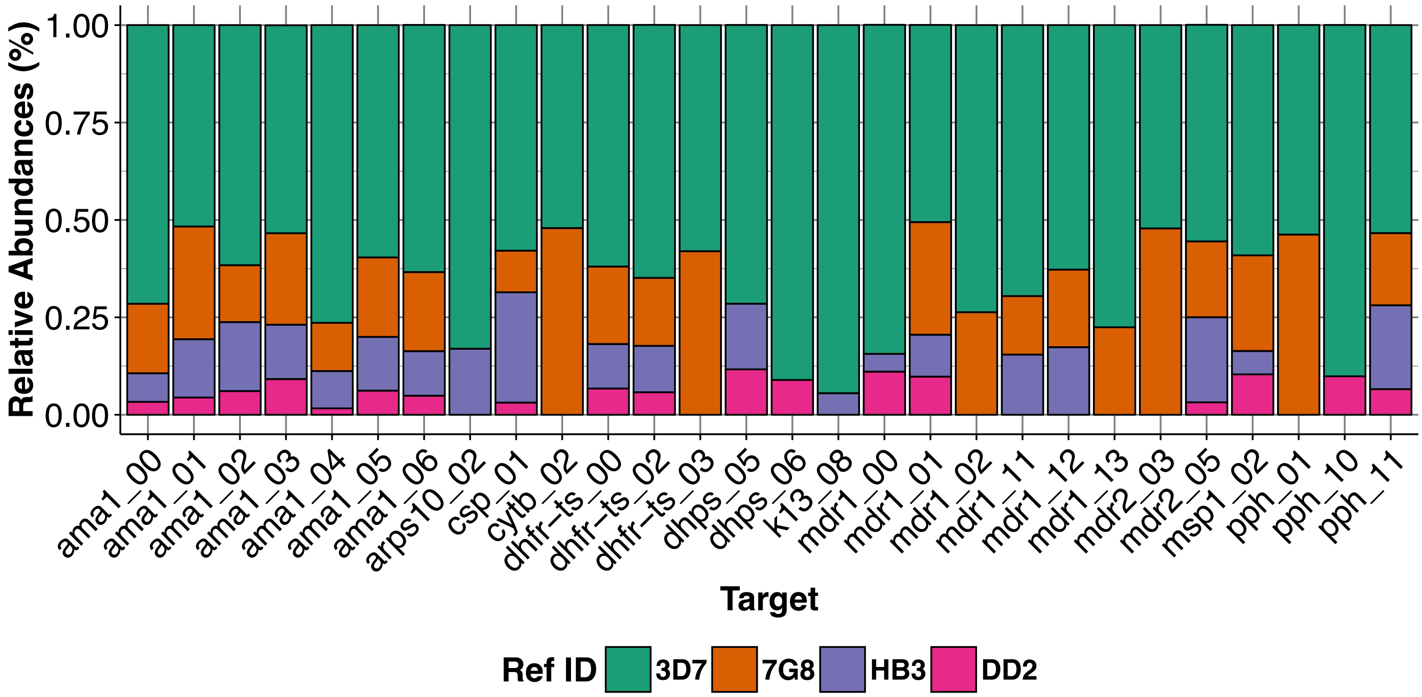

Figure S9

Haplotype Recovery of Simulation Data - Platform

The average haplotype recovery of the simulation datasets binned on technology and minor haplotype divergence for each program. The top row shows the average haplotype recovery of the minor haplotypes closely related to the major haplotype (**Figure S4a**), and the bottom is the average haplotype recovery of minor haplotypes close to another minor haplotype (**Figure S4c**). Error bars represent one standard error.

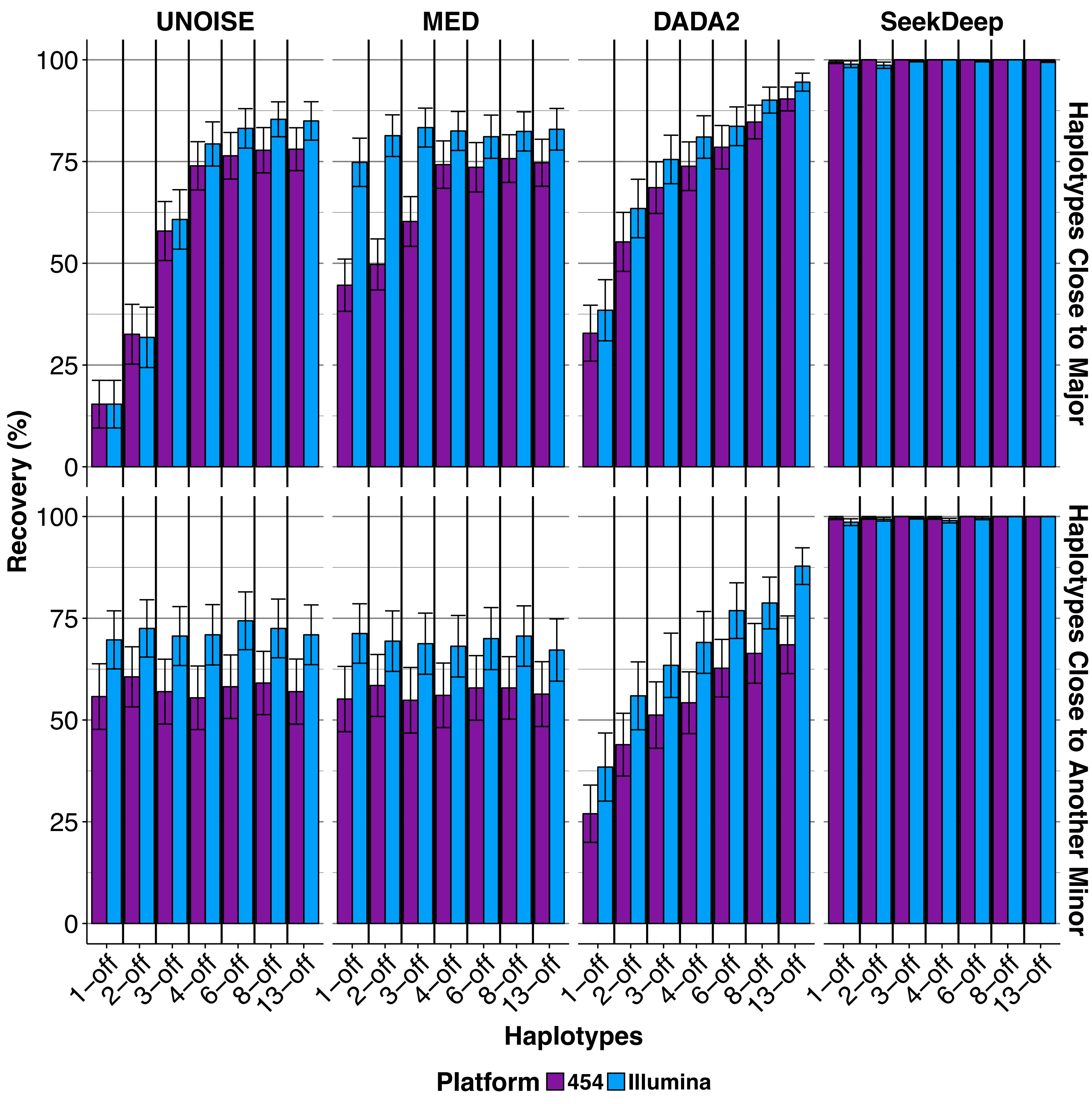

Figure S10

Haplotype Recovery of Simulation Data - Read Depth

The average haplotype recovery of the simulation datasets binned on simulated read depth and haplotype divergence for each program. The top row shows the average haplotype recovery of the minor haplotypes closely related to the major haplotype (**Figure S4a**) and the bottom is the average haplotype recovery of minor haplotypes close to another minor haplotype (**Figure S4c**). Error bars represent standard error.

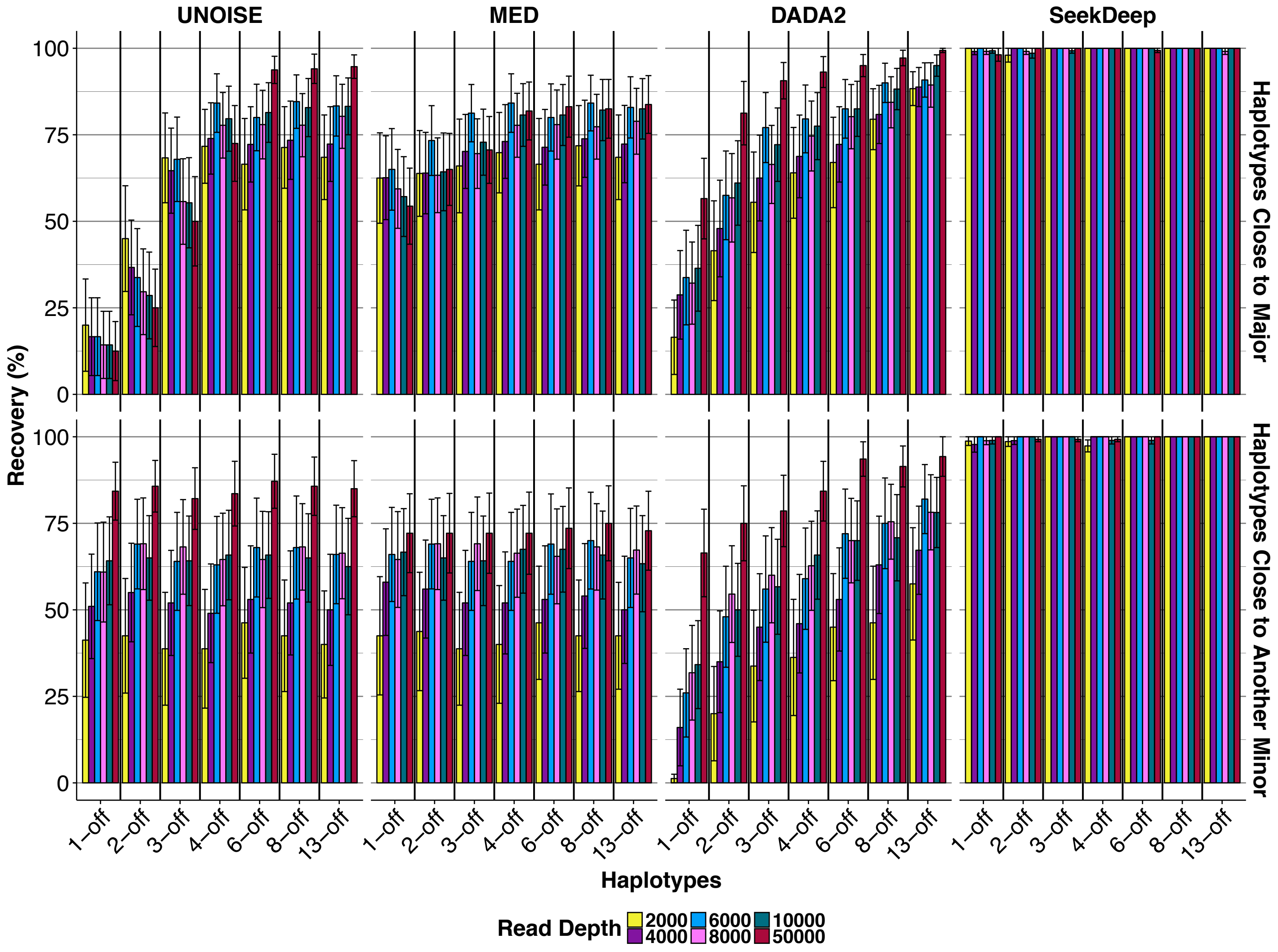

Figure S11

Haplotype Recovery of Simulation Data - Minor Haplotype Abundance

The average haplotype recovery of the simulation datasets binned on minor haplotype abundance and divergence for each program. The top row shows the average haplotype recovery of the minor haplotypes close to a major haplotype (**Figure S4a**), and the bottom is the average haplotype recovery of minor haplotypes close to another minor haplotype (**Figure S4c**). Error bars represent standard error.

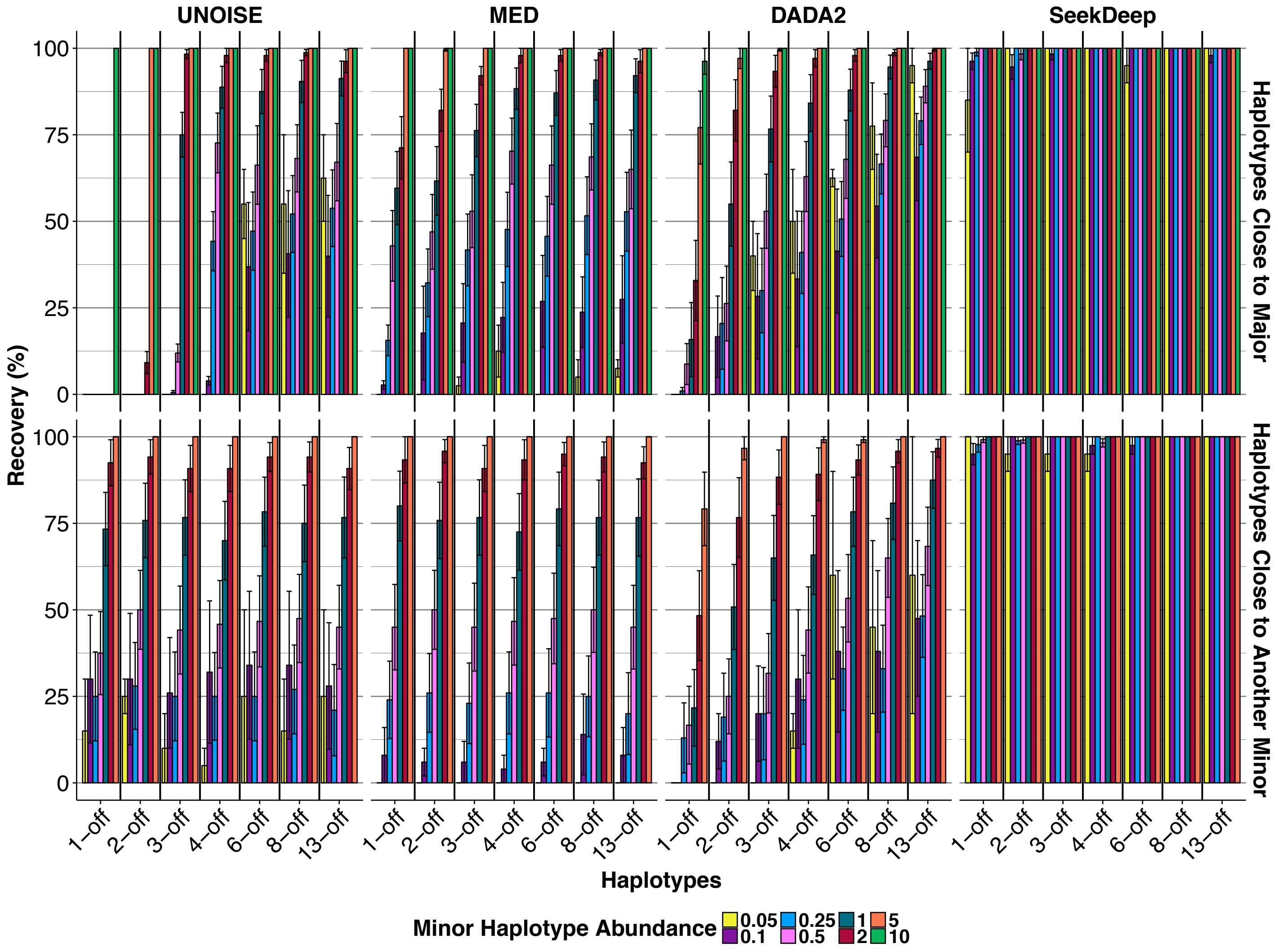

Figure S12

Predicted vs Expected Haplotype Abundances for Simulations

Panel **a)** is the plot of every simulated minor haplotype comparing each program's predicted abundance to the expected abundance based on direct read counts (**Figure S4** mixtures). Panel **b)** is the complementary plot of the major haplotypes for all simulations. **c)** A violin plot of the root mean squared error (RMSE) on the y-axis on a log scale for each program for all simulated datasets. For panels **a)** and **b),** the black line of identity for expected and predicted is shown. If points are above the line of identity the program is overestimating the abundance of the haplotype and if points are below the line the program is underestimating the abundance of the haplotype. The Spearman's correlation (R^2) is in the upper left corner of each plot.

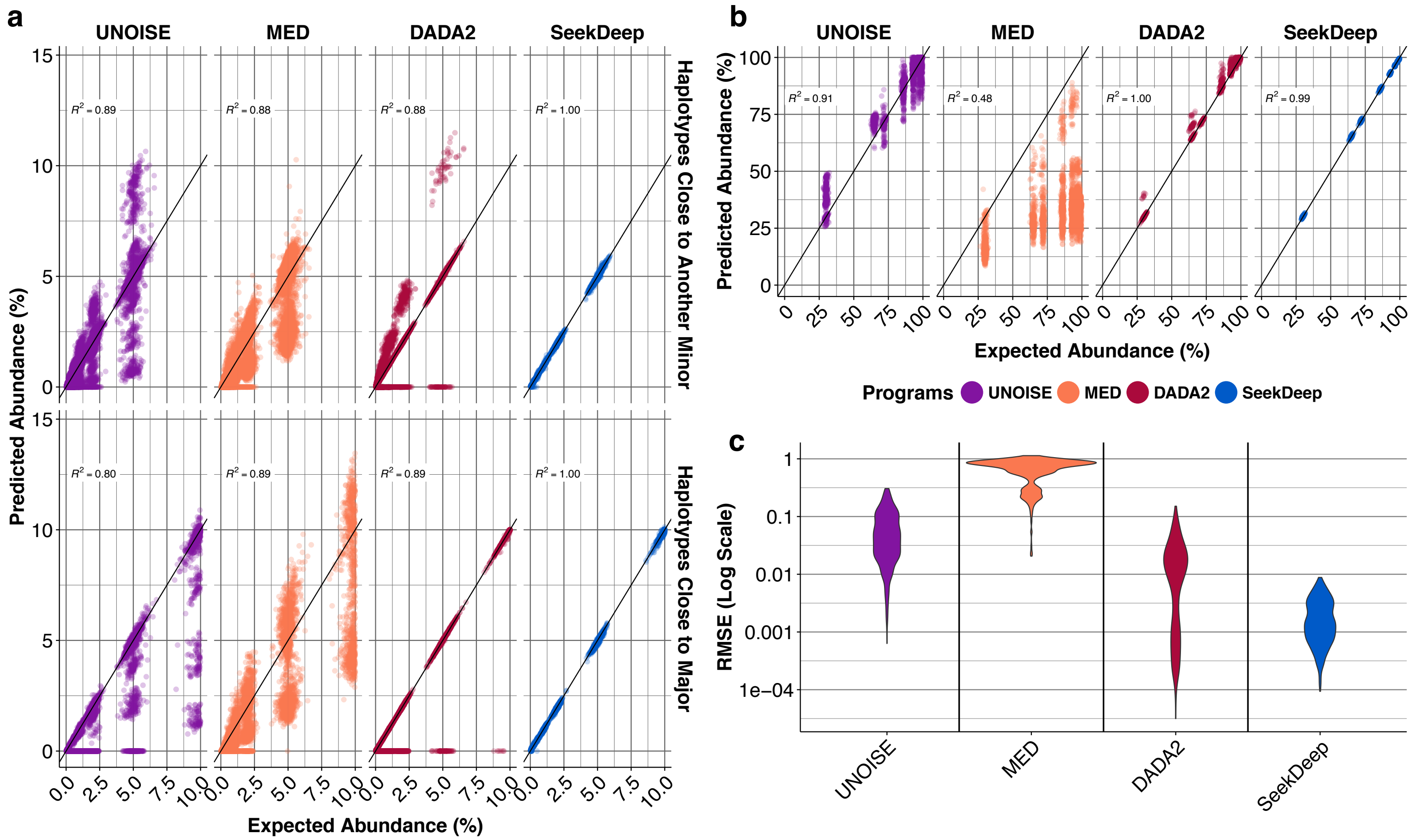

Figure S13

Predicted vs Expected Haplotype Abundances for Simulations of Closely Related Haplotypes

The predicted vs expected abundances for known haplotypes differing by only one (**Panels a-b**), two (**Panels c-d**), or three (**Panels e-f**) bases is plotted to illustrate the effects of different read depths and technology for each program. Data points are colored by program. A diagonal black line is drawn to indicate perfect predicted for the expected abundance. Points above this line are overestimating haplotype abundance and points below this line are underestimating haplotype abundance. The Spearman's correlation has been placed in the upper left corner of each plot. Panels are **a)** haplotypes one mismatch off a major haplotype, **b)** haplotypes one mismatch off of another minor haplotype, **c)** haplotypes two mismatches off a major haplotype, **d)** haplotypes two mismatches off of another minor haplotype, **e)** haplotypes three mismatches off a major haplotype, and **f)** haplotypes three mismatches off of another minor haplotype.

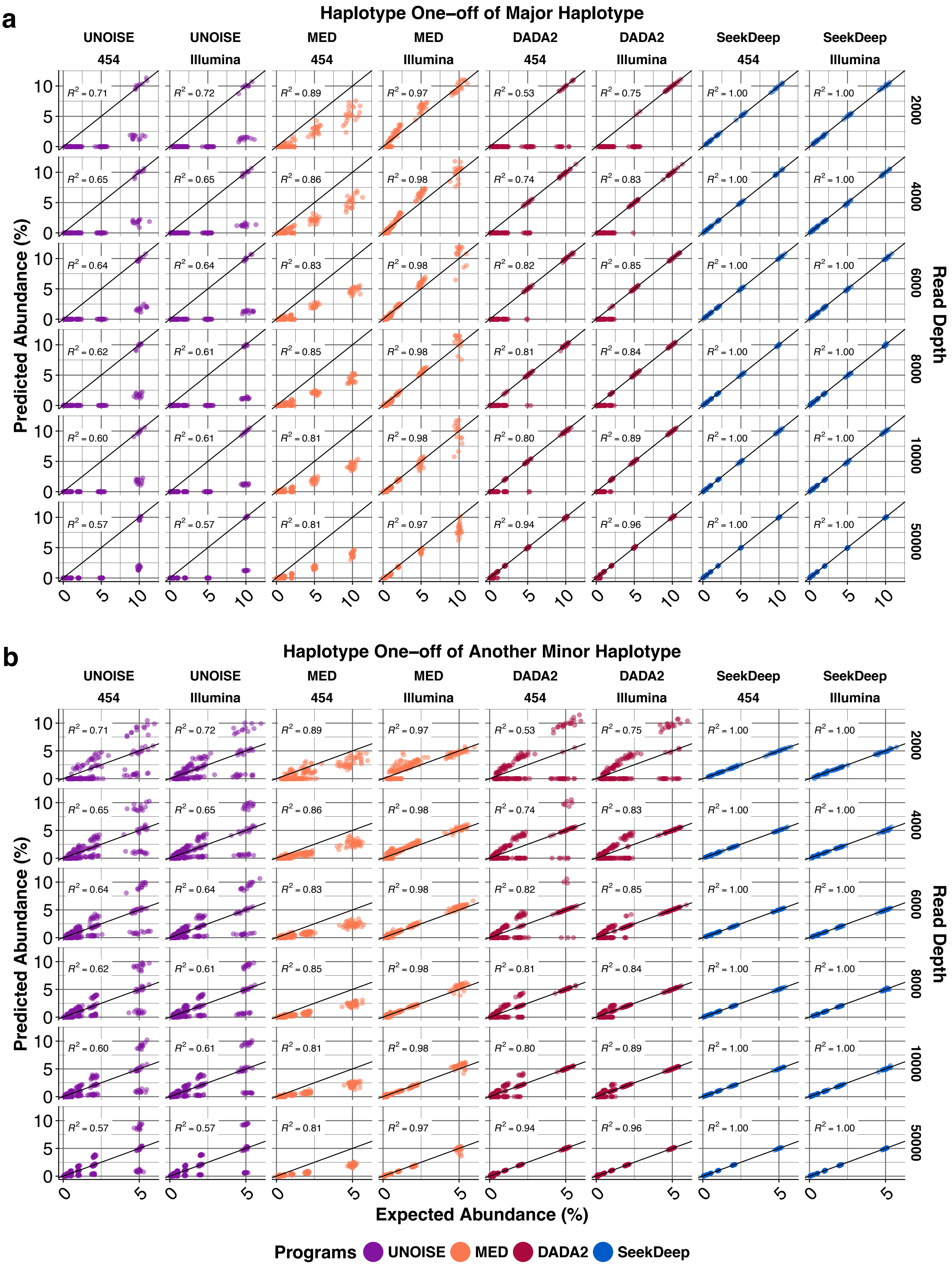
a
Haplotype One−off of Major Haplotype
UNOISE 454
UNOISE Illumina
MED 454
MED Illumina
DADA2 454
DADA2 Illumina
SeekDeep 454
SeekDeep Illumina
Read Depth
2000
4000
6000
8000
10000
5000
Predicted Abundance (%)
b
Haplotype One−off of Another Minor Haplotype
Expected Abundance (%)
Programs
UNOISE
MED
DADA2
SeekDeep

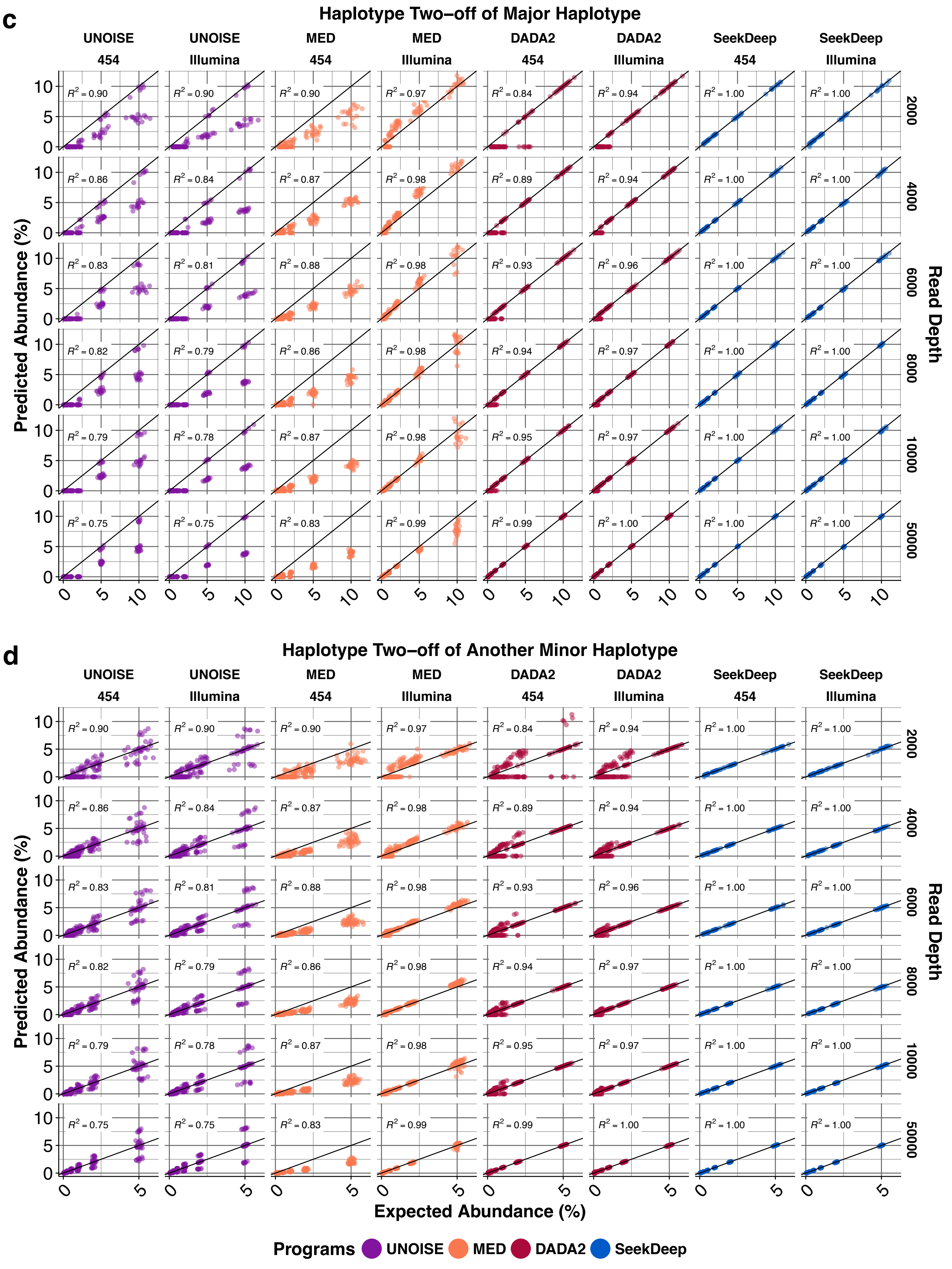
c
Haplotype Two−off of Major Haplotype
UNOISE 454
UNOISE Illumina
MED 454
MED Illumina
DADA2 454
DADA2 Illumina
SeekDeep 454
SeekDeep Illumina
2000: R^2 = 0.90, R^2 = 0.90, R^2 = 0.90, R^2 = 0.97, R^2 = 0.84, R^2 = 0.94, R^2 = 1.00, R^2 = 1.00
4000: R^2 = 0.86, R^2 = 0.84, R^2 = 0.87, R^2 = 0.98, R^2 = 0.89, R^2 = 0.94, R^2 = 1.00, R^2 = 1.00
6000: R^2 = 0.83, R^2 = 0.81, R^2 = 0.88, R^2 = 0.98, R^2 = 0.93, R^2 = 0.96, R^2 = 1.00, R^2 = 1.00
8000: R^2 = 0.82, R^2 = 0.79, R^2 = 0.86, R^2 = 0.98, R^2 = 0.94, R^2 = 0.97, R^2 = 1.00, R^2 = 1.00
10000: R^2 = 0.79, R^2 = 0.78, R^2 = 0.87, R^2 = 0.98, R^2 = 0.95, R^2 = 0.97, R^2 = 1.00, R^2 = 1.00
50000: R^2 = 0.75, R^2 = 0.75, R^2 = 0.83, R^2 = 0.99, R^2 = 0.99, R^2 = 1.00, R^2 = 1.00, R^2 = 1.00
Predicted Abundance (%)
Read Depth
d
Haplotype Two−off of Another Minor Haplotype
UNOISE 454
UNOISE Illumina
MED 454
MED Illumina
DADA2 454
DADA2 Illumina
SeekDeep 454
SeekDeep Illumina
2000: R^2 = 0.90, R^2 = 0.90, R^2 = 0.90, R^2 = 0.97, R^2 = 0.84, R^2 = 0.94, R^2 = 1.00, R^2 = 1.00
4000: R^2 = 0.86, R^2 = 0.84, R^2 = 0.87, R^2 = 0.98, R^2 = 0.89, R^2 = 0.94, R^2 = 1.00, R^2 = 1.00
6000: R^2 = 0.83, R^2 = 0.81, R^2 = 0.88, R^2 = 0.98, R^2 = 0.93, R^2 = 0.96, R^2 = 1.00, R^2 = 1.00
8000: R^2 = 0.82, R^2 = 0.79, R^2 = 0.86, R^2 = 0.98, R^2 = 0.94, R^2 = 0.97, R^2 = 1.00, R^2 = 1.00
10000: R^2 = 0.79, R^2 = 0.78, R^2 = 0.87, R^2 = 0.98, R^2 = 0.95, R^2 = 0.97, R^2 = 1.00, R^2 = 1.00
50000: R^2 = 0.75, R^2 = 0.75, R^2 = 0.83, R^2 = 0.99, R^2 = 0.99, R^2 = 1.00, R^2 = 1.00, R^2 = 1.00
Predicted Abundance (%)
Read Depth
Expected Abundance (%)
Programs UNOISE MED DADA2 SeekDeep

e

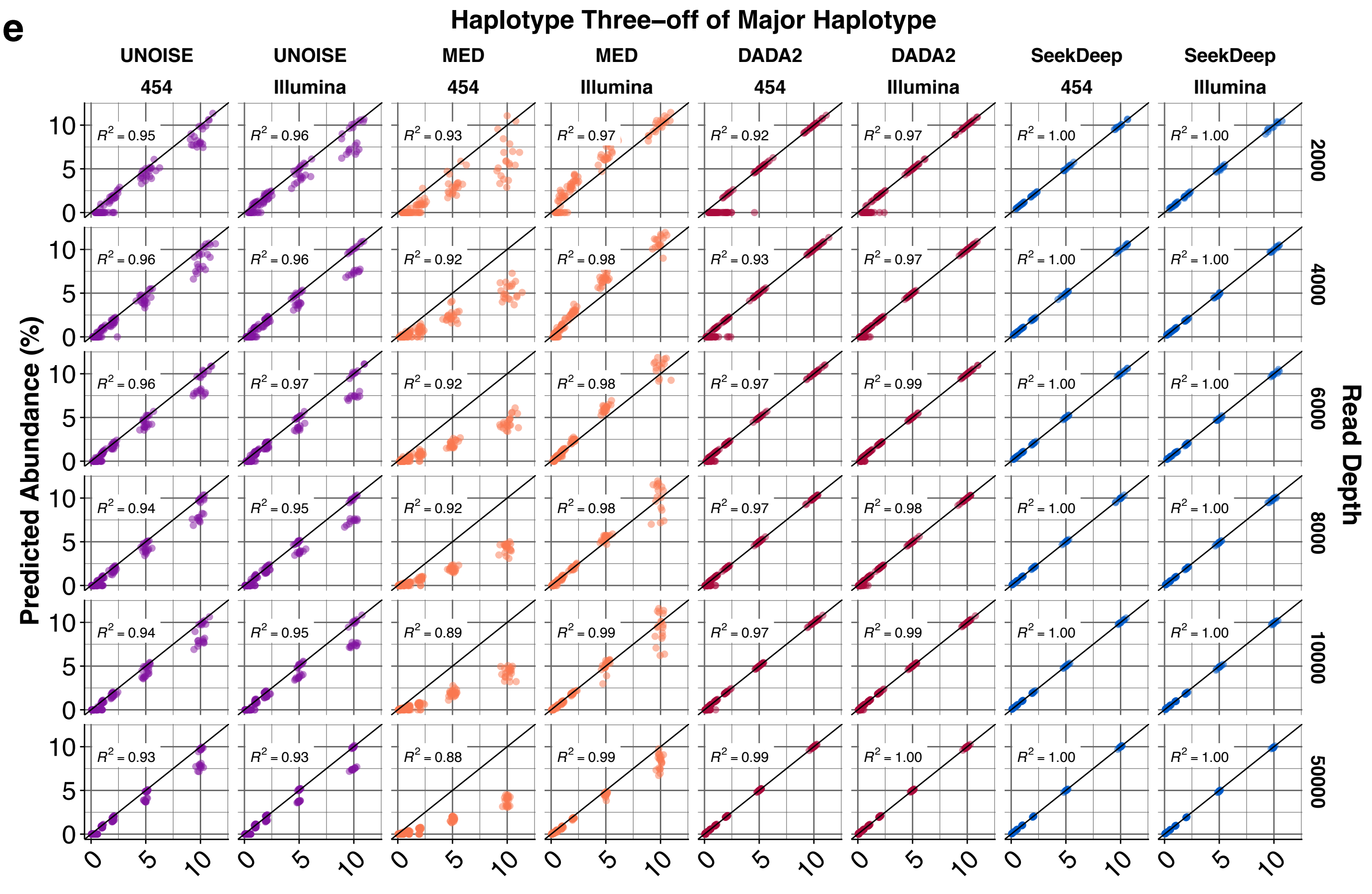
Haplotype Three–off of Major Haplotype
UNOISE 454
UNOISE Illumina
MED 454
MED Illumina
DADA2 454
DADA2 Illumina
SeekDeep 454
SeekDeep Illumina
Predicted Abundance (%)
Read Depth
2000
4000
6000
8000
10000
50000
$R^2 = 0.95$ $R^2 = 0.96$ $R^2 = 0.93$ $R^2 = 0.97$ $R^2 = 0.92$ $R^2 = 0.97$ $R^2 = 1.00$ $R^2 = 1.00$
$R^2 = 0.96$ $R^2 = 0.96$ $R^2 = 0.92$ $R^2 = 0.98$ $R^2 = 0.93$ $R^2 = 0.97$ $R^2 = 1.00$ $R^2 = 1.00$
$R^2 = 0.96$ $R^2 = 0.97$ $R^2 = 0.92$ $R^2 = 0.98$ $R^2 = 0.97$ $R^2 = 0.99$ $R^2 = 1.00$ $R^2 = 1.00$
$R^2 = 0.94$ $R^2 = 0.95$ $R^2 = 0.92$ $R^2 = 0.98$ $R^2 = 0.97$ $R^2 = 0.98$ $R^2 = 1.00$ $R^2 = 1.00$
$R^2 = 0.94$ $R^2 = 0.95$ $R^2 = 0.89$ $R^2 = 0.99$ $R^2 = 0.97$ $R^2 = 0.99$ $R^2 = 1.00$ $R^2 = 1.00$
$R^2 = 0.93$ $R^2 = 0.93$ $R^2 = 0.88$ $R^2 = 0.99$ $R^2 = 0.99$ $R^2 = 1.00$ $R^2 = 1.00$ $R^2 = 1.00$

f

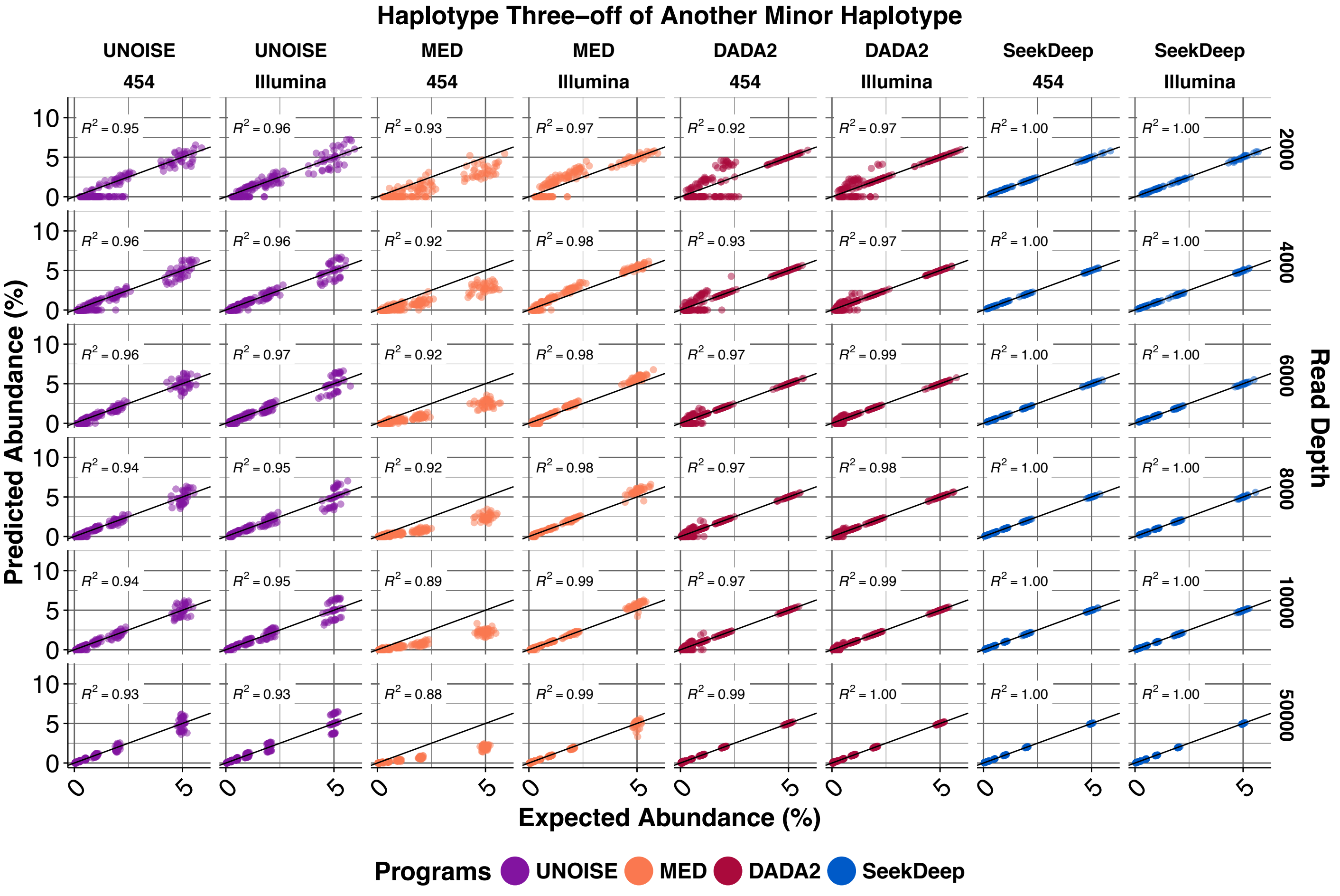
Haplotype Three–off of Another Minor Haplotype
UNOISE 454
UNOISE Illumina
MED 454
MED Illumina
DADA2 454
DADA2 Illumina
SeekDeep 454
SeekDeep Illumina
Predicted Abundance (%)
Read Depth
2000
4000
6000
8000
10000
50000
$R^2 = 0.95$ $R^2 = 0.96$ $R^2 = 0.93$ $R^2 = 0.97$ $R^2 = 0.92$ $R^2 = 0.97$ $R^2 = 1.00$ $R^2 = 1.00$
$R^2 = 0.96$ $R^2 = 0.96$ $R^2 = 0.92$ $R^2 = 0.98$ $R^2 = 0.93$ $R^2 = 0.97$ $R^2 = 1.00$ $R^2 = 1.00$
$R^2 = 0.96$ $R^2 = 0.97$ $R^2 = 0.92$ $R^2 = 0.98$ $R^2 = 0.97$ $R^2 = 0.99$ $R^2 = 1.00$ $R^2 = 1.00$
$R^2 = 0.94$ $R^2 = 0.95$ $R^2 = 0.92$ $R^2 = 0.98$ $R^2 = 0.97$ $R^2 = 0.98$ $R^2 = 1.00$ $R^2 = 1.00$
$R^2 = 0.94$ $R^2 = 0.95$ $R^2 = 0.89$ $R^2 = 0.99$ $R^2 = 0.97$ $R^2 = 0.99$ $R^2 = 1.00$ $R^2 = 1.00$
$R^2 = 0.93$ $R^2 = 0.93$ $R^2 = 0.88$ $R^2 = 0.99$ $R^2 = 0.99$ $R^2 = 1.00$ $R^2 = 1.00$ $R^2 = 1.00$
Expected Abundance (%)
Programs UNOISE MED DADA2 SeekDeep

Figure S14

False Haplotype Abundances from Simulations

The relative abundances of predicted false haplotypes are binned by read depth (x-axis), technology (columns), and the use of replicates (rows). The y-axis is log scaled and is the relative abundance at which the false haplotypes were predicted. See **Table S2** for exact number of false haplotypes.

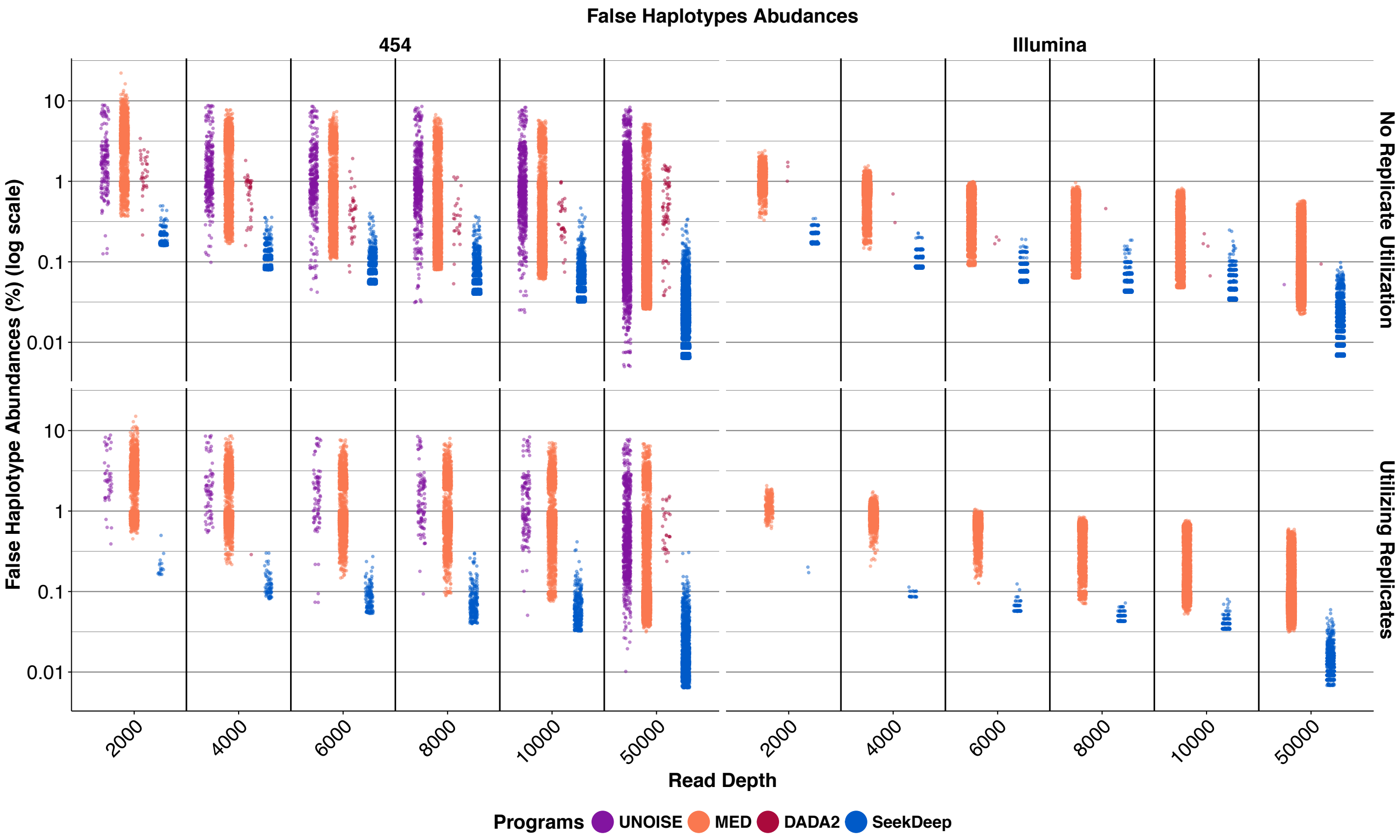

Figure S15

***In vitro* P. falciparum Illumina Mixtures Performance**

The expected vs predicted abundances for all the target regions for when any of the programs failed to recover one of the expected reference haplotypes. The leftmost bar is the expected abundance based on direct mapping to the known reference and subsequent bars represent predicted abundances by program.

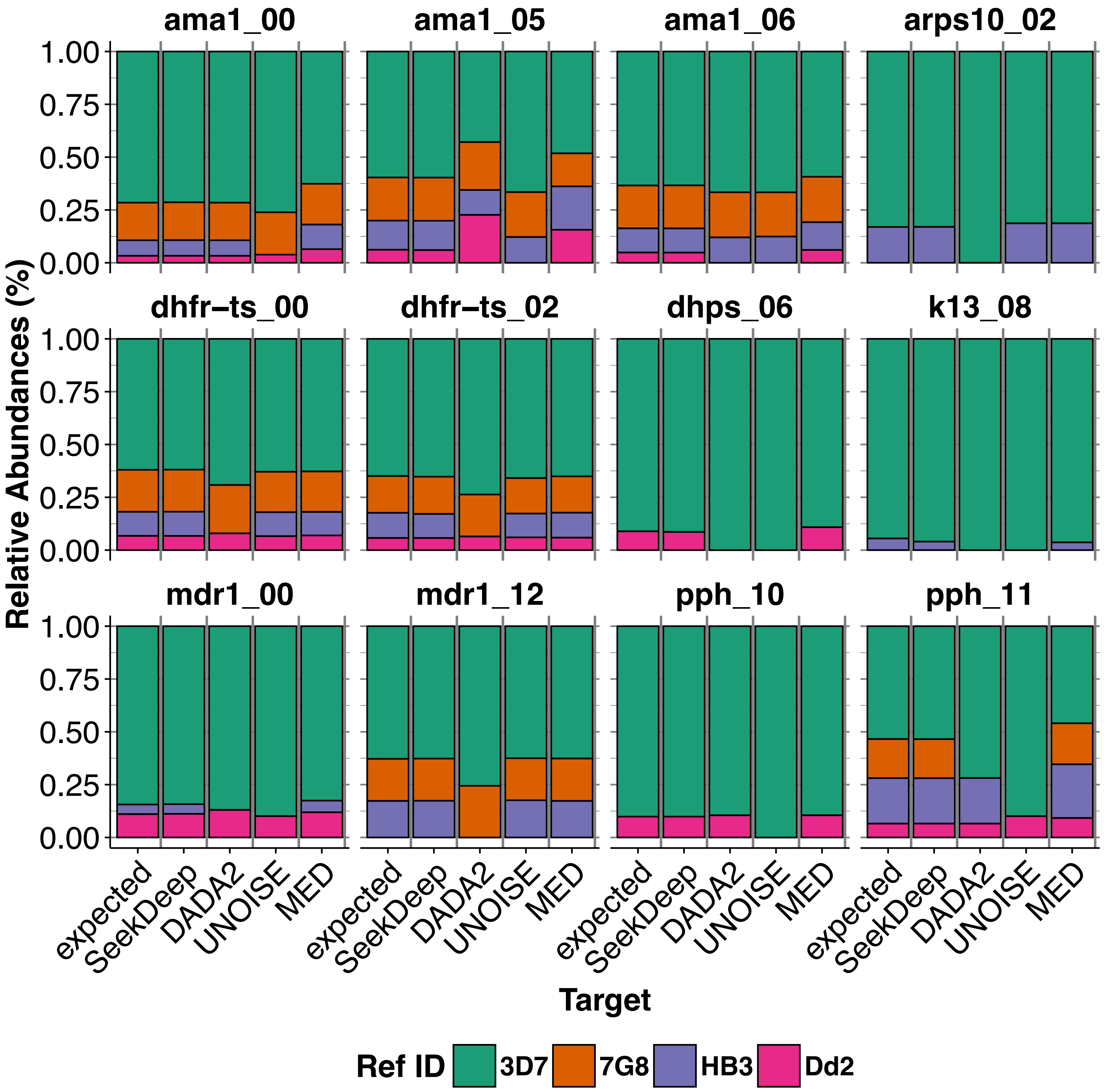

Figure S16

Down-sampled Mock Microbiome Haplotype Recovery of Haplotypes Differing by One Base

a) Shows the haplotype recovery of the 23 haplotypes that are one-off from another haplotype in an overall mixture of 47 bacterial haplotypes which were down-sampled from the Salipante *et al*. 2014 data. Each of the three original replicates was down sampled randomly 10 times for each of 10 different read depths, which means each read depth has 30 randomly down sampled samples. **b)** A bar graph of the greatest observed abundance of missed one-off haplotype is shown for each program at each read depth.

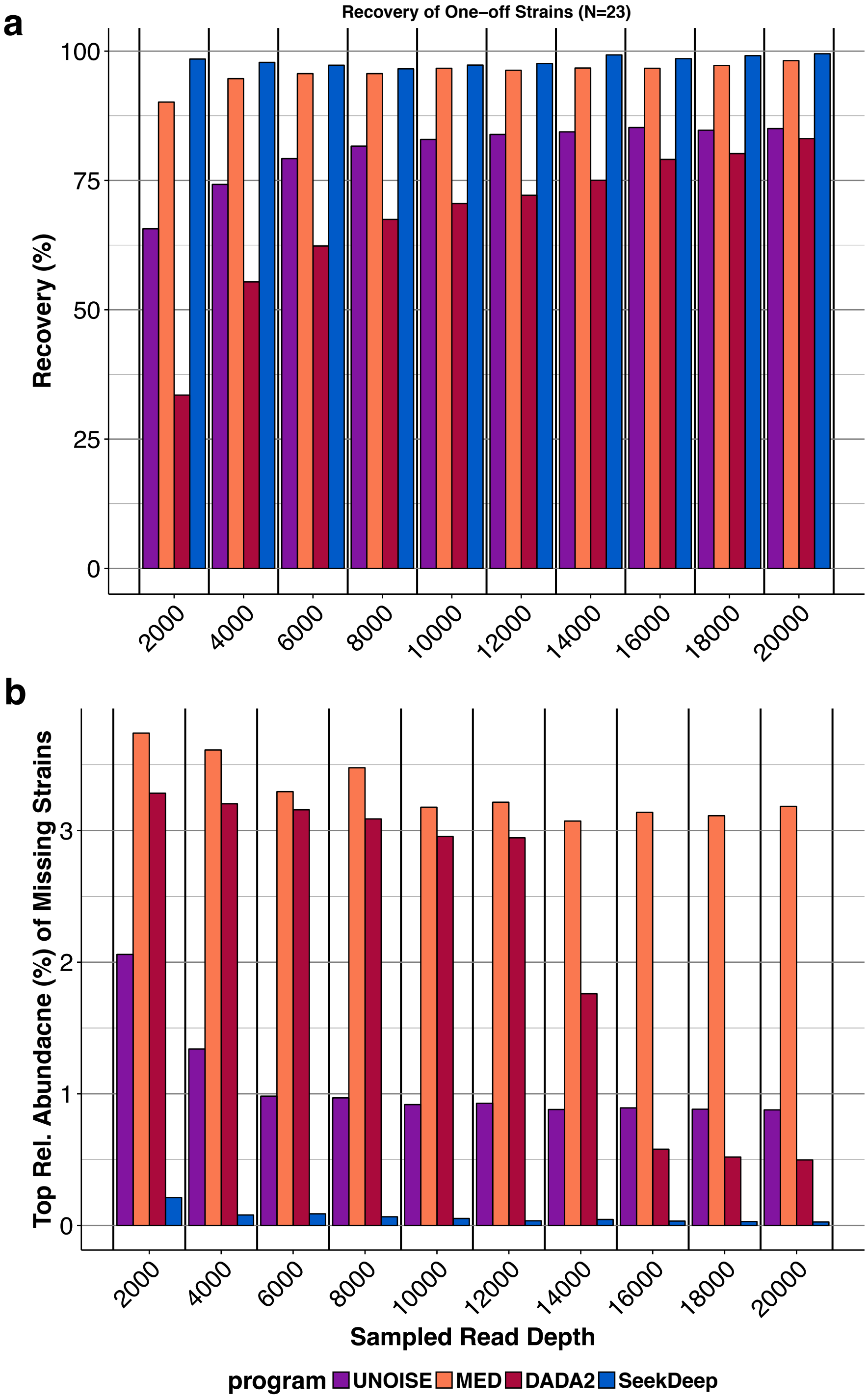

Figure S17

Down-sampled Mock Microbiome Predicted vs Expected Haplotype Abundances

a) A plot of predicted vs expected haplotype abundances is shown for each program for all down sampled datasets from Salipante *et al*. 2014. If the program predicted an abundance that equalled the expected abundance, points would fall on the depicted black line of identity. If a program overestimated the haplotype abundance, points would fall above the line. If a program underestimated the haplotype abundance, points would fall below the line. **b)** The log-scaled RMSE is shown as a violin plot for all down sampled data.

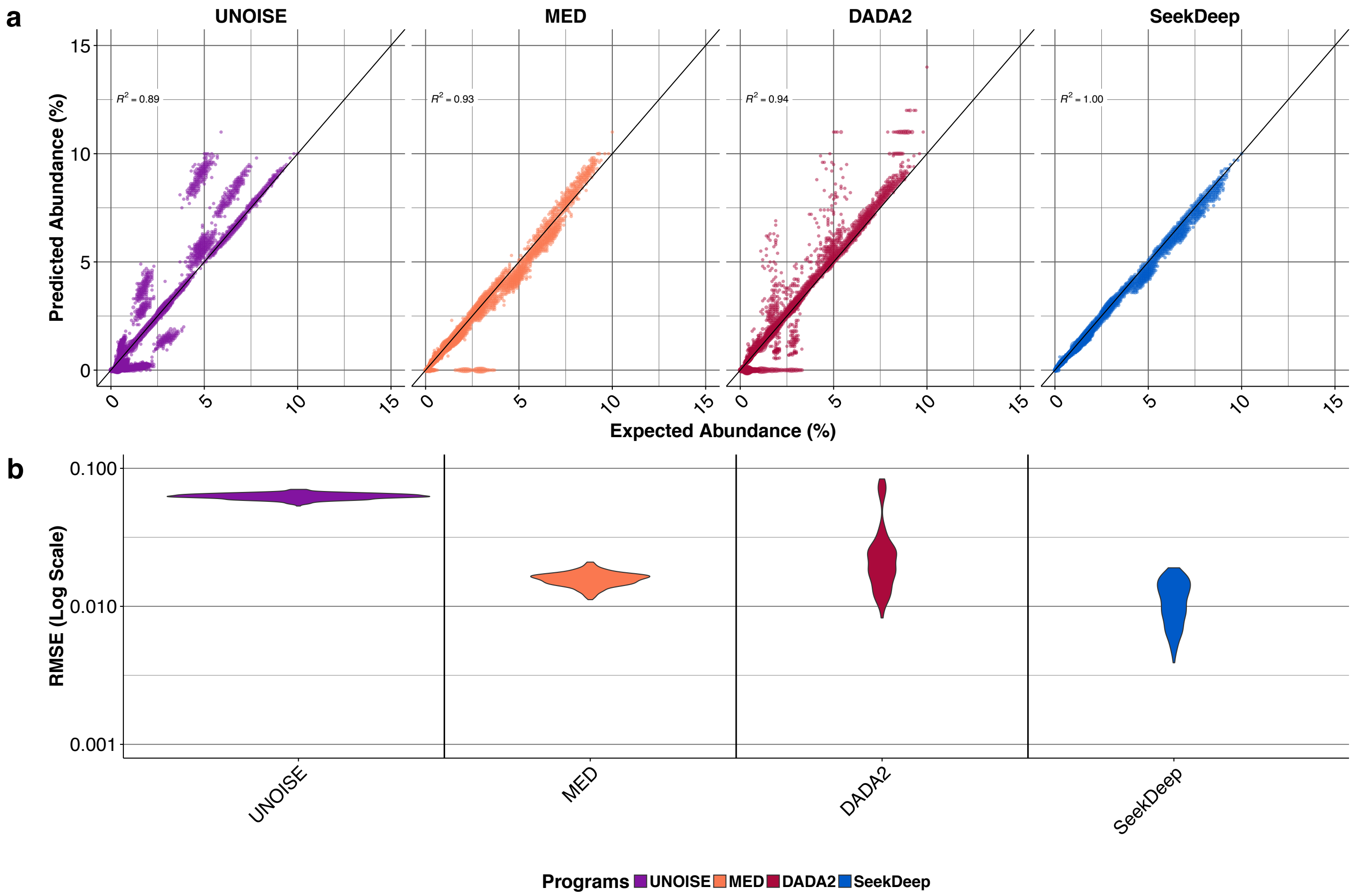

Figure S18 - In Vitro EBV Illumina Performance

a) The mean haplotype recovery for the EBV datasets. **b)** Predicted abundance (y-axis) estimated by the various programs is plotted against the expected abundance (x-axis). Deviation from the line of identity represents the error and is summarized by the correlation coefficient. **c)** False haplotypes are shown on a jitterplot to demonstrate their relative abundances and numbers (see **Table S6** for exact counts). Results are shown per program and also by the effect of utilizing or not utilizing replicates (haplotypes are only accepted if they appear in both replicates).

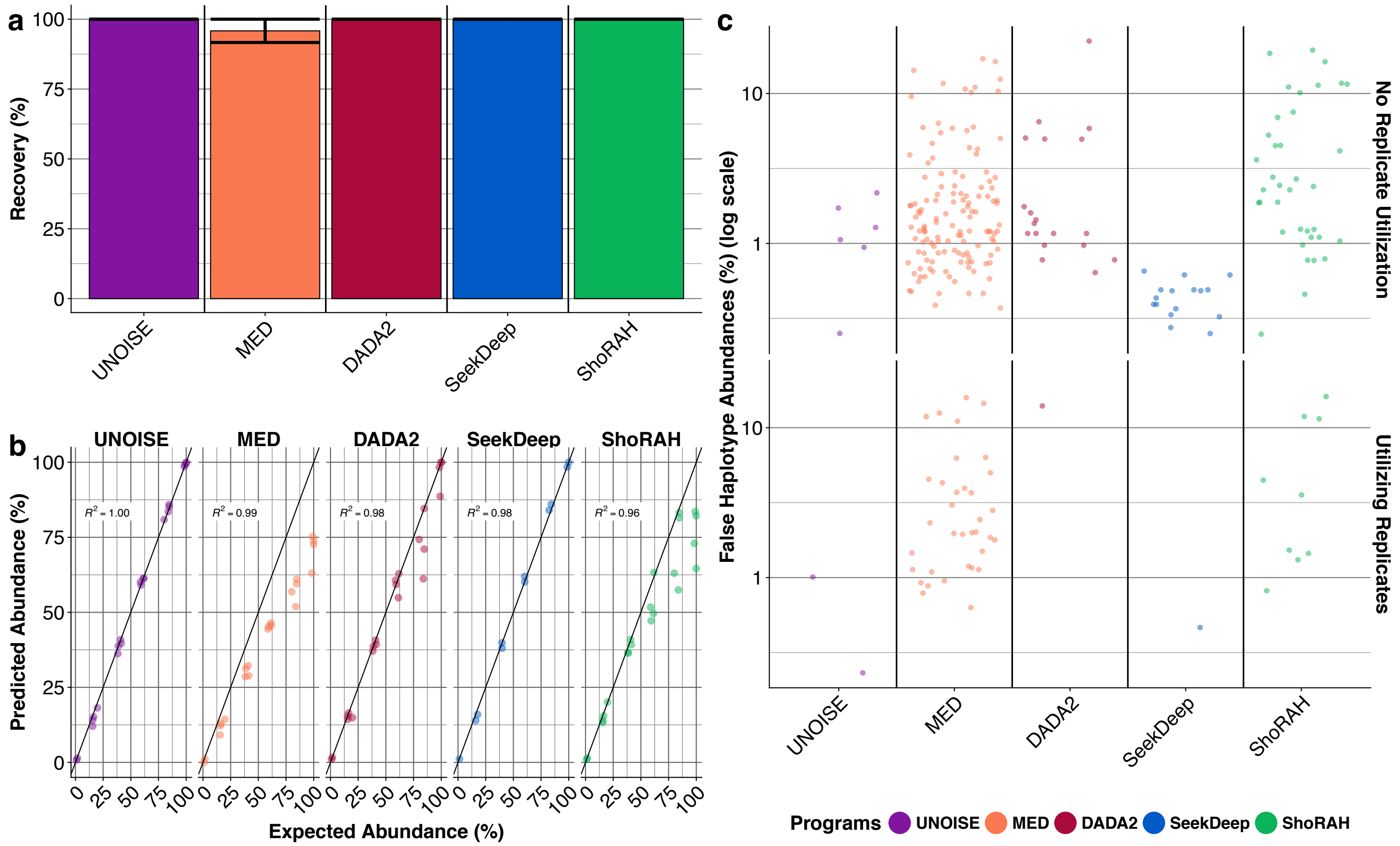

Figure S19 - In Vitro HIV Illumina Performance

a) The mean haplotype recovery for the HIV datasets. **b)** Predicted abundance (y-axis) estimated by the various programs is plotted against the expected abundance (x-axis). Deviation from the line of identity represents the error and is summarized by the correlation coefficient. **c)** False haplotypes are shown on a jitterplot to demonstrate their relative abundances and numbers (see **Table S7** for exact counts). Results are shown per program and also by the effect of utilizing or not utilizing replicates (haplotypes are only accepted if they appear in both replicates).

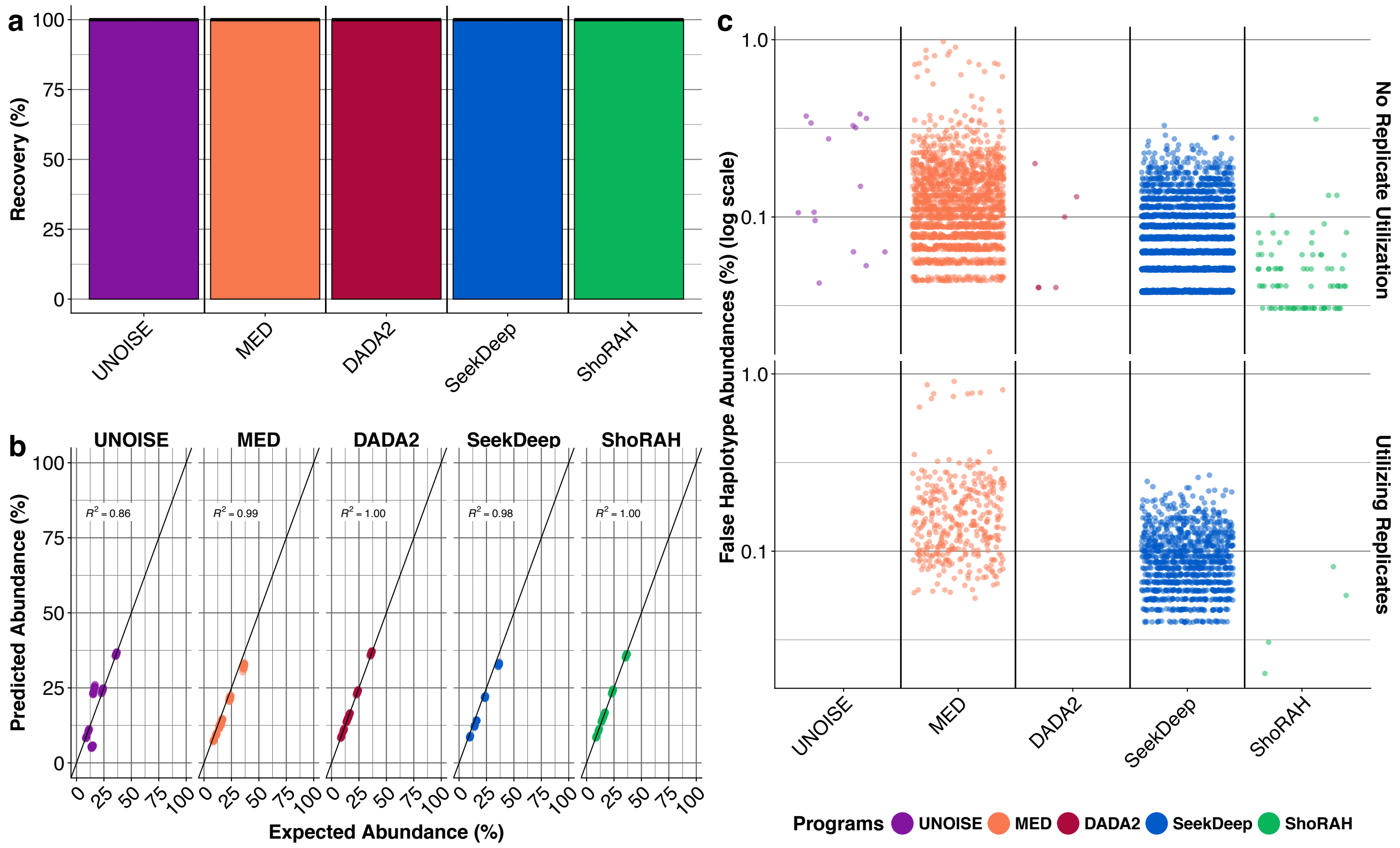

Figure S20

Chimera Detection

a) A jitter scatter plot of the SeekDeep results for the haplotypes for samples that had appreciable chimeras with the x-axis being sample and y-axis being predicted relative abundance (truncated at 10%, all haplotype above 10% are true haplotypes). The haplotypes are to appear in both replicates to be plotted. The haplotypes that were marked chimeric are orange diamonds, true haplotypes less than 10% are green circles, and false haplotypes that didn't get marked chimeric are grey circles. **b)** A plot comparing the the predicted relative abundances of the replicates for the false haplotypes which demonstrates the reproducibility of chimera formation across PCR reactions. Deviation from the line of identity represents the difference in the replicates and is summarized by the correlation coefficient.

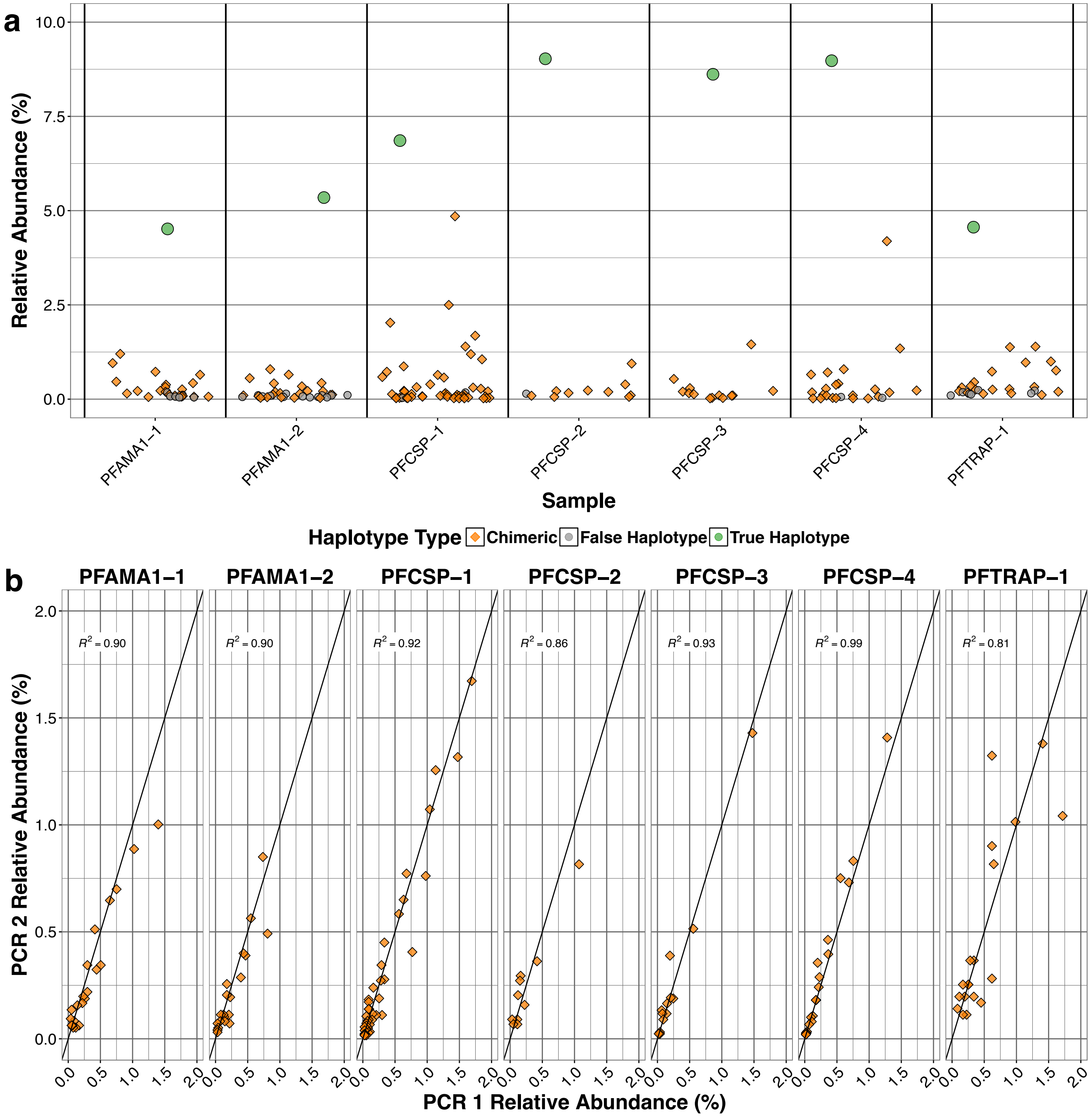

Figure S21

OTU Clustering Performance on Simulation Data

SeekDeep offers OTU clustering that is based on only high quality differences rather than any difference. This helps to improve both haplotype recovery and false haplotype creation compared to the OTU clustering offered by USEARCH. Performance of OTU clustering for SeekDeep is shown for both 99% and 97% OTU clustering while only 97% OTU clustering is shown from USEARCH due to a reported bug in the program that only allows 97% clustering. **a)** Haplotype recovery is shown for the two different simulation mixtures depicted in **Figure S2**. Haplotype recovery is binned by the degree of difference (and corresponding percent identity) between the haplotypes, and is further stratified by read depth. **b)** A jitter scatter plot is shown of the relative abundance of false haplotypes, stratified by read-depth (x-axis) and sequencing technology. Bars and points are colored by program and OTU level of clustering.

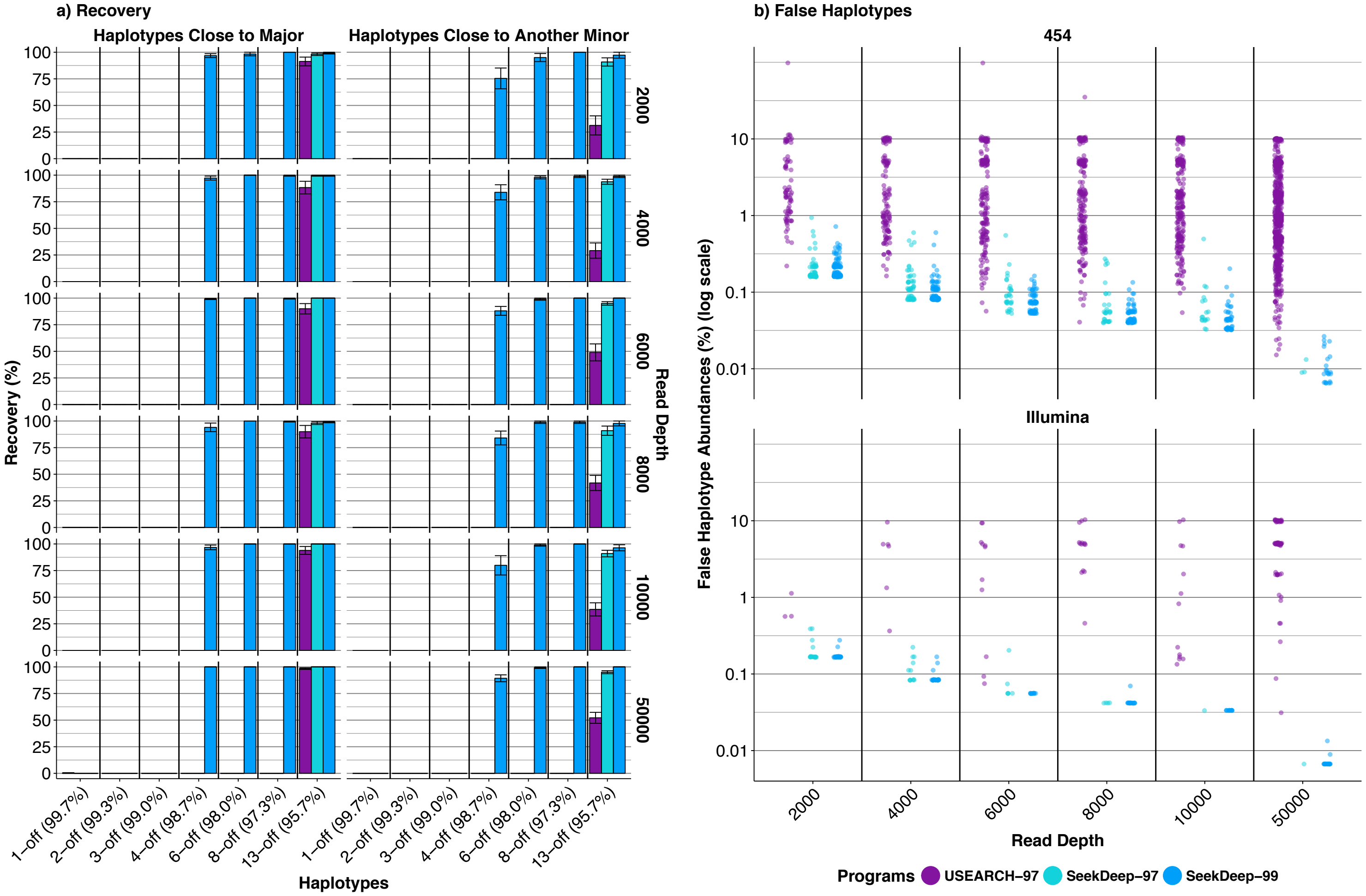

Figure S22

Collapsing on Single-base Differences Performance on Simulation Data

SeekDeep, like swarm, can be tuned to account for the number of differences upon which to collapse; however, unlike swarm, SeekDeep can account for type and quality of errors during clustering. Here, we demonstrate the performance of swarm collapsing on 1 difference compared against SeekDeep collapsing on 1 high quality difference and allowing for low abundance and low quality differences as well is shown. **a)** Haplotype recovery is shown for the two different simulation mixtures depicted in **Figure S4**. It is binned by the degree of difference (and corresponding percent identity) between the haplotypes, and is further stratified by read depth. **b)** A jitter scatter plot of the relative abundance of false haplotypes, stratified by read-depth (x-axis) and sequencing technology. Bars and points are colored by program.

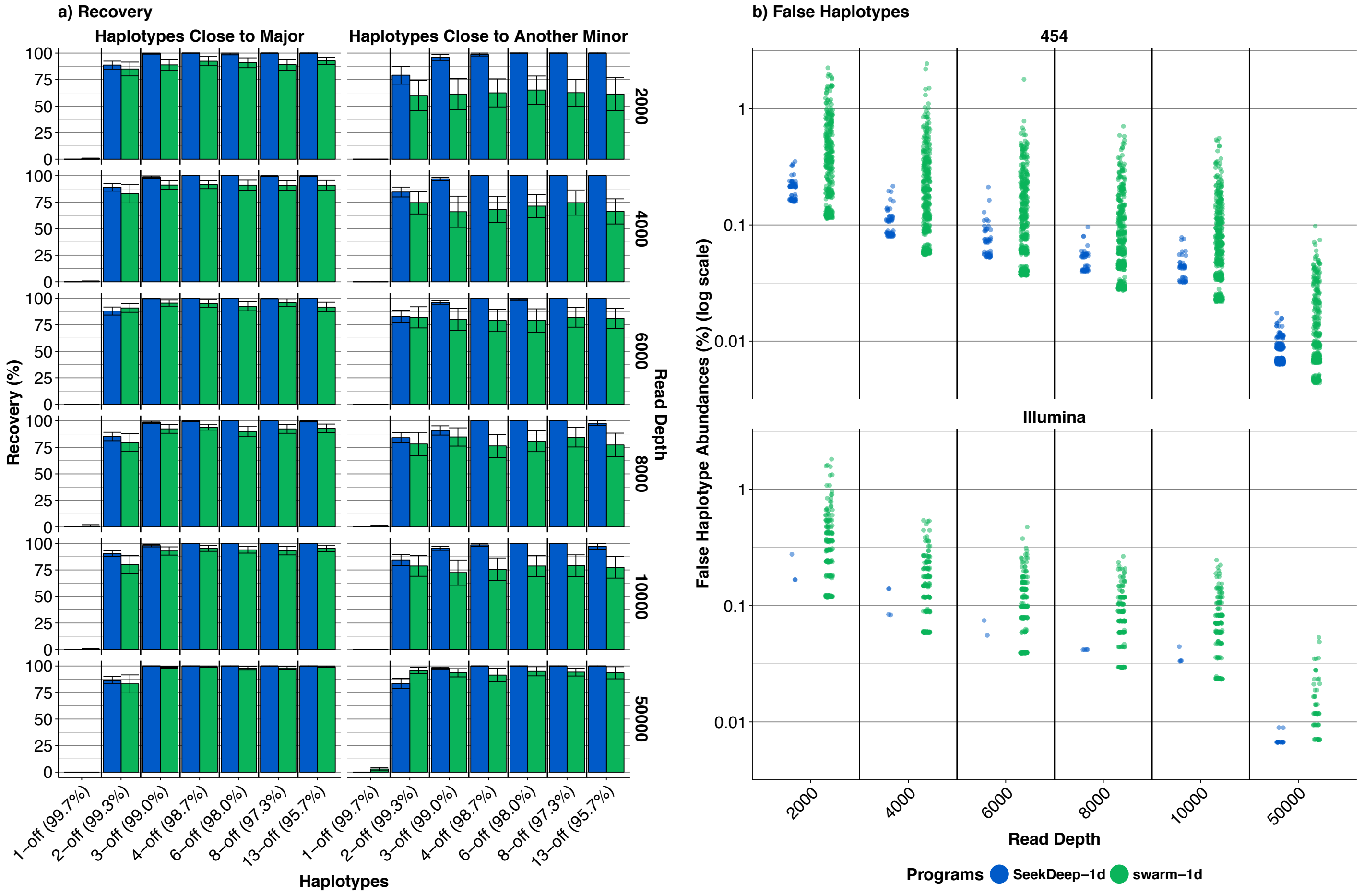

Figure S23

Program Run Times

The distribution of program run times in seconds is shown **a)** for simulation datasets across read depths, **b)** for all the randomly down sampled samples from Salipante *et al*. 2014, **c)** for the *in vitro P. falciparum* control datasets. These times should approximate what a user would expect using a personal computer.

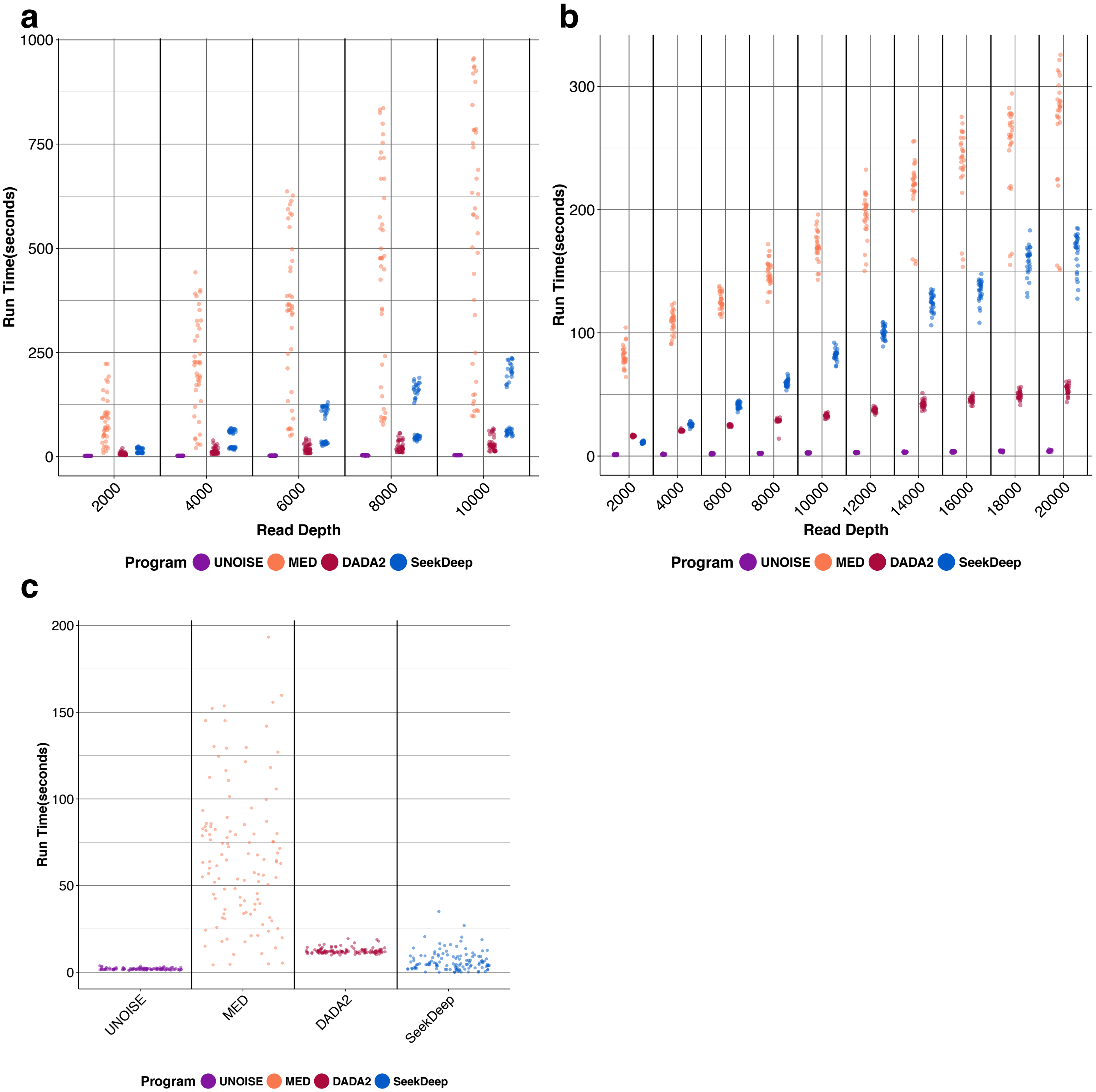

Figure S24

Haplotype Recovery of Expected Haplotypes and Creation of False Haplotype above ≥0.25% on Simulated Datasets

Performance on the simulations dataset is shown, using a minimum haplotype abundance threshold of 0.25%. **a)** SeekDeep is able to haplotype recovery all expected haplotypes across all read depths simulated. **b)** By making a cut at 0.25% SeekDeep calls practically no false haplotypes. Detection of haplotypes at ≥ 0.25% likely approaches what can be detected by sampling and PCR. At this level of resolution SeekDeep has excellent performance characteristics with better haplotype recovery than other programs with minimal false haplotypes rates that occur only at low abundances. See **Table S8** for exact number of false haplotypes.

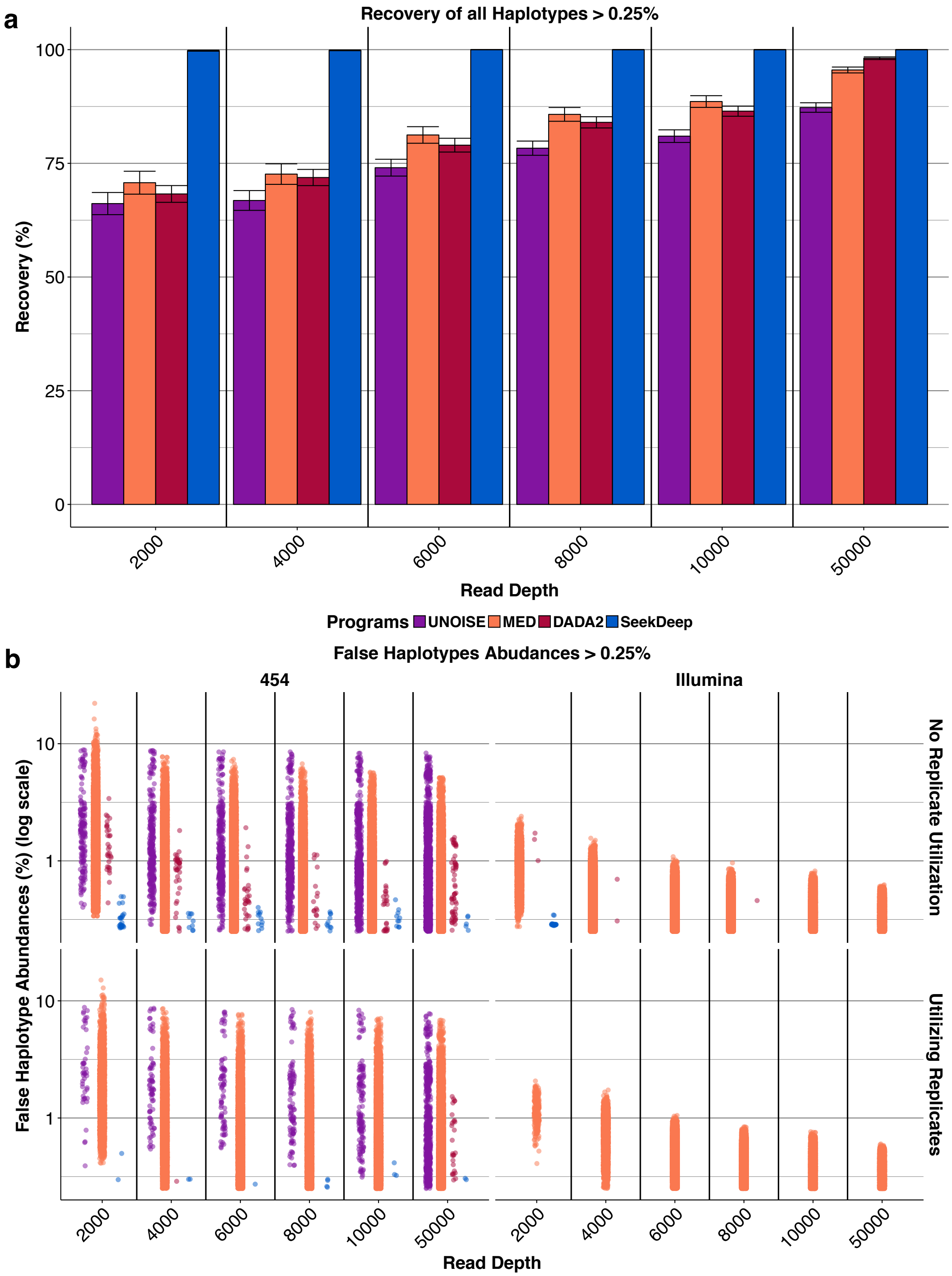

Supplement: Supplementary Data [file gkx1201_supp.zip › nar-00215-met-k-2017-File006.pdf]
