# Supplementary material for: SeekDeep: single-base resolution de novo clustering for amplicon deep sequencing
Source: Nucleic Acids Res. 2017 Nov 30;46(4):e21. doi: 10.1093/nar/gkx1201 (PMC5829576; doi:10.1093/nar/gkx1201)
Supplement: Supplementary Data [file gkx1201_supp.zip › nar-00215-met-k-2017-File008.html]

Program Run Commands


# Program Run Commands

#### *Nicholas Hathaway*

- MED
  - 454/Ion Torrent
  - Illumina
- DADA2
  - Parired end reads
  - Single end reads
- SeekDeep
  - 454/Iont Torrent
  - Illumina
- UNOISE

# MED

Used version 2.1

## 454/Ion Torrent

For 454/Ion Torrent MED paper suggest aligning reads first to a reference

```
pynast -i reads.fasta -t refFile.fasta -l 320
decompose --skip-check-input-file -o decompseOut reads_pynast_aligned.fasta -H
```

## Illumina

```
decompose --skip-check-input-file -o decompseOut reads.fasta
```

# DADA2

Used version 1.0.3

## Parired end reads

The input arguments were changed appropriately for each dataset.

```
#!/usr/bin/env Rscript
suppressMessages(library(dada2)); #packageVersion("dada2")

path <- "illumina_extraction" 
filtpath <- "dada2/filt"
filePattern <- "[0-9]+.fastq$"
outputdir <- "dada2/"
trimLeft <- 20 
trimRight <- 320
on454 <- T
checkBimeras <- F


fns <- sort(list.files(path)) # Sort should keep them paired in order
fastqs <- fns[grepl(filePattern, fns)]
fnFs <- fastqs[grepl("_R1", fastqs)]
fnRs <- fastqs[grepl("_R2", fastqs)]
sam_names <- sapply(strsplit(fnFs, "_"), `[`, 1)


filtFs <- paste0(filtpath, sapply(strsplit(fnFs, "\\."), `[`, 1), "_filt.fastq.gz")
filtRs <- paste0(filtpath, sapply(strsplit(fnRs, "\\."), `[`, 1), "_filt.fastq.gz")
for(i in seq_along(fnFs)) {
  fastqPairedFilter(paste0(path, c(fnFs[i], fnRs[i])), c(filtFs[i], filtRs[i]), maxN=0, maxEE=2, truncQ=2, compress=TRUE, verbose=TRUE)
}

derepFs <- derepFastq(filtFs, verbose=TRUE)
derepRs <- derepFastq(filtRs, verbose=TRUE)

#names(derepFs) <- sam_names
#names(derepRs) <- sam_names

dadaFs.part <- dada(derepFs, err=inflateErr(tperr1,3), selfConsist = TRUE)
dadaRs.part <- dada(derepRs, err=inflateErr(tperr1,3), selfConsist = TRUE)

errF <- dadaFs.part$err_out
errR <- dadaRs.part$err_out

dadaFs <- dada(derepFs, err=errF, pool = TRUE, selfConsist=FALSE) # 9m
dadaRs <- dada(derepRs, err=errR, pool = TRUE, selfConsist=FALSE) # 6m

mergers <- mergePairs(dadaFs, derepFs, dadaRs, derepRs)


seqtab.all <- makeSequenceTable(mergers)
bim <- isBimeraDenovo(seqtab.all,minFoldParentOverAbundance = 3,  verbose=TRUE)
seqtab <- seqtab.all[,!bim]

chr <- function(n) { rawToChar(as.raw(n)) }
writeFastqFromDada<-function(dadaSingleSample, filename, bim){
  fastqLines = c()
  sampleName = basename(filename)
  for (seqNum in 1:length(dadaSingleSample$sequence)){
    if(!missing(bim)){
      if(bim[names(bim) == dadaSingleSample$sequence[seqNum]]){
        fastqLines = c(fastqLines, as.character(paste("@CHI_", sampleName, ".", as.character(seqNum), "_t", as.character(dadaSingleSample$clustering$abundance[seqNum]), sep = "")))
      }else{
        fastqLines = c(fastqLines, as.character(paste("@", sampleName, ".", as.character(seqNum), "_t", as.character(dadaSingleSample$clustering$abundance[seqNum]), sep = "")))
      }
      fastqLines = c(fastqLines,  as.character(dadaSingleSample$sequence[seqNum]))
      fastqLines = c(fastqLines,"+")
      print (dadaSingleSample$quality)
      fastqLines = c(fastqLines,as.character(chr(round(dadaSingleSample$quality + 33)[seqNum,])))
    }else{
      fastqLines = c(fastqLines, as.character(paste("@", sampleName, ".", as.character(seqNum), "_t", as.character(dadaSingleSample$clustering$abundance[seqNum]), sep = "")))
      fastqLines = c(fastqLines,  as.character(dadaSingleSample$sequence[seqNum]))
      fastqLines = c(fastqLines,"+")
      fastqLines = c(fastqLines,as.character(chr(round(dadaSingleSample$quality + 33)[seqNum,])))
    }
  }
  fileConn<-file(filename)
  writeLines(fastqLines, fileConn)
  close(fileConn)
}
writeFastaFromDadaMerged<-function(dadaSingleSample, filename, bim){
  fastqLines = c()
  sampleName = basename(filename)
  for (seqNum in 1:length(dadaSingleSample$sequence)){
    if(!missing(bim)){
      if(bim[names(bim) == dadaSingleSample$sequence[seqNum]]){
        fastqLines = c(fastqLines, as.character(paste(">CHI_", sampleName, ".", as.character(seqNum), "_t", as.character(dadaSingleSample$abundance[seqNum]), sep = "")))
      }else{
        fastqLines = c(fastqLines, as.character(paste(">", sampleName, ".", as.character(seqNum), "_t", as.character(dadaSingleSample$abundance[seqNum]), sep = "")))
      }
      fastqLines = c(fastqLines,  as.character(dadaSingleSample$sequence[seqNum]))
    }else{
      fastqLines = c(fastqLines, as.character(paste(">", sampleName, ".", as.character(seqNum), "_t", as.character(dadaSingleSample$abundance[seqNum]), sep = "")))
      fastqLines = c(fastqLines,  as.character(dadaSingleSample$sequence[seqNum]))
    }
  }
  fileConn<-file(filename)
  writeLines(fastqLines, fileConn)
  close(fileConn)
}

currentBimHaps = bim
writeFastaFromDadaMerged(mergers, paste0(outputdir, "dada2_", "V1MID7-n10000-1", ".fasta"), currentBimHaps)
```

## Single end reads

The input arguments were changed appropriately for each dataset.

```
#!/usr/bin/env Rscript
suppressMessages(library(dada2)); #packageVersion("dada2")

path <- "454_extraction" 
filePat <- "[0-9]+.fastq$"
outputdir <- "dada2/"
trimLeft <- 20 
trimRight <- 320
on454 <- T
checkBimeras <- F


chr <- function(n) { rawToChar(as.raw(n)) }
writeFastqFromDada<-function(dadaSingleSample, filename, bim){
  fastqLines = c()
  sampleName = basename(filename)
  for (seqNum in 1:length(dadaSingleSample$sequence)){
    if(!missing(bim)){
      if(bim[names(bim) == dadaSingleSample$sequence[seqNum]]){
        fastqLines = c(fastqLines, as.character(paste("@CHI_", sampleName, ".", as.character(seqNum), "_t", as.character(dadaSingleSample$clustering$abundance[seqNum]), sep = "")))
      }else{
        fastqLines = c(fastqLines, as.character(paste("@", sampleName, ".", as.character(seqNum), "_t", as.character(dadaSingleSample$clustering$abundance[seqNum]), sep = "")))
      }
      fastqLines = c(fastqLines,  as.character(dadaSingleSample$sequence[seqNum]))
      fastqLines = c(fastqLines,"+")
      fastqLines = c(fastqLines,as.character(chr(round(dadaSingleSample$quality + 33)[seqNum,])))
    }else{
      fastqLines = c(fastqLines, as.character(paste("@", sampleName, ".", as.character(seqNum), "_t", as.character(dadaSingleSample$clustering$abundance[seqNum]), sep = "")))
      fastqLines = c(fastqLines,  as.character(dadaSingleSample$sequence[seqNum]))
      fastqLines = c(fastqLines,"+")
      fastqLines = c(fastqLines,as.character(chr(round(dadaSingleSample$quality + 33)[seqNum,])))
    }
  }
  fileConn<-file(filename)
  writeLines(fastqLines, fileConn)
  close(fileConn)
}
runDada<-function(inputPath,  outputDir, filePattern, trimLeft, trimRight, on454){
  fns <- list.files(inputPath)
  fastqs <- fns[grepl(filePattern, fns)]
  sample_names <- sapply(strsplit(fastqs, ".",fixed = T), `[`, 1)
  fastqs <- paste0(inputPath, fastqs)
  # Make filenames for the filtered fastq files
  filtFs <- paste0(outputDir, sample_names, "_filt.fastq.gz")
  # Filter
  for(i in seq_along(fastqs)) {
    fastqFilter(fastqs[i], filtFs[i],
                trimLeft=trimLeft, truncLen=trimRight, 
                maxN=0, maxEE=Inf, truncQ=0, 
                compress=TRUE, verbose=TRUE)
  }
  dereps <- derepFastq(filtFs, verbose=TRUE)
  # Name the derep-class objects by the sample names
  if(length(filtFs) > 1){
    names(dereps) <- sample_names
  }
  if(on454){
    dadaFsBoth <- dada(dereps, err=inflateErr(tperr1,3), selfConsist = TRUE, HOMOPOLYMER_GAP_PENALTY=-1, BAND_SIZE=32)
  }else{
    dadaFsBoth <- dada(dereps, err=inflateErr(tperr1,3), selfConsist = TRUE)
  }
  return(dadaFsBoth)
}

output = runDada(path,  outputdir, filePat, trimLeft, trimRight, on454)
if (checkBimeras) {
  seqtab.all <- makeSequenceTable(output)
  bim <- isBimeraDenovo(seqtab.all, verbose=TRUE)
  if(class(output)[1] == "list"){
    for (samp in names(output)){
      currentSampHaps = seqtab.all[rownames(seqtab.all) == samp, ]
      currentBimHaps = bim[(1:length(currentSampHaps))[currentSampHaps > 0]]
      writeFastqFromDada(output[[samp]], paste0(outputdir, "dada2_", samp, ".fastq"), currentBimHaps)
    }
  }else{
    fns <- list.files(path)
    fastqs <- fns[grepl(filePat, fns)]
    sample_names <- sapply(strsplit(fastqs, ".",fixed = T), `[`, 1)
    currentSampHaps = seqtab.all[1, ]
    currentBimHaps = bim[(1:length(currentSampHaps))[currentSampHaps > 0]]
    writeFastqFromDada(output, paste0(outputdir, "dada2_", sample_names[1], ".fastq"), currentBimHaps)
  }
  
} else {
  if(class(output)[1] == "list"){
    for (samp in names(output)){
      writeFastqFromDada(output[[samp]], paste0(outputdir, "dada2_", samp, ".fastq"))
    }
  }else{
    fns <- list.files(path)
    fastqs <- fns[grepl(filePat, fns)]
    sample_names <- sapply(strsplit(fastqs, ".",fixed = T), `[`, 1)
    writeFastqFromDada(output, paste0(outputdir, "dada2_", sample_names[1], ".fastq"))
  }
}
```

# SeekDeep

Version used 2.4.0  
See http://baileylab.umassmed.edu/SeekDeep for more detailed usage but below are some generic run commands

## 454/Iont Torrent

```
#extraction
SeekDeep extractor --fastq reads.fastq --id idFile.txt --multiplex --minlen 320  --maxLen 350 
#clustering per sample
SeekDeep qluster --fastq reads.fastq --ionTorrent 
#population clustering 
SeekDeep processClusters --fastq output.fastq --strictErrors
```

## Illumina

```
#extraction
SeekDeep extractor --fastq reads.fastq --id idFile.txt --multiplex --minlen 320  --maxLen 350 -qualCheckCutOff .89 -qualCheck 30
#clustering per sample
SeekDeep qluster --fastq reads.fastq --illumina 
#population clustering 
SeekDeep processClusters --fastq output.fastq --strictErrors
```

# UNOISE

Version 9.2

```
usearch9.2 -fastq_filter reads.fastq -fastq_maxee 2.5 -fastaout filter.fa -relabel Filt -log filter.log 
usearch9.2 -fastx_uniques filter.fa -sizeout -relabel Uniq -fastaout uniques.fa -log derep.log
usearch9.2 -unoise2 uniques.fa -fastaout denoised.fa -log unoise.log -minampsize 3
usearch9.2 -usearch_global reads.fastq -db denoised.fa -strand plus -id 0.99 -log make_otutab.log -otutabout otutab_den.txt -biomout otutab_den.json -mothur_shared_out otutab_den.mothur
```
